# Structural and Spectroscopic Insights into Catalytic Intermediates of a [NiFe]‐hydrogenase from Group 3

**DOI:** 10.1002/cbic.202500692

**Published:** 2025-10-13

**Authors:** Marion Jespersen, Christian Lorent, Olivier N. Lemaire, Ingo Zebger, Tristan Wagner

**Affiliations:** ^1^ Max Planck Institute for Marine Microbiology Celsiusstraße 1 28359 Bremen Germany; ^2^ Institut für Chemie Technische Universität Berlin Straße des 17. Juni 135 10623 Berlin Germany; ^3^ Institut de Biologie Structurale Université Grenoble Alpes, CEA, CNRS 38000 Grenoble France; ^4^ Present address: Monash University, Clayton Melbourne 3800 Australia

**Keywords:** electron paramagnetic resonance spectroscopy, F_420_‐reducing [NiFe]‐hydrogenase, hydrogen activation, thermophilic methanogen, vibrational spectroscopy, X‐ray crystallography

## Abstract

Hydrogenases catalyze reversible H_2_ production and are potential models for renewable energy catalysts. Here, the full redox landscape of a group 3 [NiFe]‐hydrogenase from methanothermococcus thermolithotrophicus is elucidated, resembling group 1 enzymes. Structural and spectroscopic analyses reveal a catalytic‐ready state with nickel seesaw coordination, enabling intermediate trapping and advancing mechanistic understanding of oxygen‐sensitive [NiFe] enzymes.

## Graphical abstract

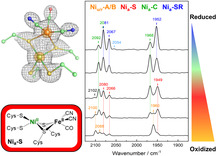

## 1 Introduction

Molecular dihydrogen (H_2_) has a great potential as a clean, renewable, and high‐energy‐density fuel. However, industrial H_2_ production requires expensive and noble metals like platinum.^[^ ^1^, ^2^ ^]^ H_2_ production catalyzed by [NiFe]‐hydrogenases offers a biological and sustainable solution for H_2_ cycling using abundant base metals, with conversion rates comparable to platinum electrodes.^[^ ^3^ ^]^ Their use in biotechnological applications requires a comprehensive knowledge of the structural and mechanistic factors that determine their reactivity. It has recently been shown that F_420_‐reducing hydrogenases (FRH, belonging to group 3a hydrogenases) are interesting models to dissect the most oxidized catalytic redox state (Ni_a_‐S) and would therefore be a suitable blueprint for the further development of sustainable synthetic H_2_‐conversion catalysts.^[^ ^4^ ^]^

FRHs catalyze the reversible H_2_ oxidation coupled to the reduction of coenzyme F_420_ (8‐hydroxy‐5‐deazaflavin, Δ*G*°′ = −10 kJ mol^−1^).^[^ ^5^ ^,^ ^6^ ^]^ F_420_ is one of the main electron carriers of methanogenic archaea, distributing reducing power to central energy metabolism and fueling anabolic pathways.^[^ ^7^ ^]^ The overall reaction requires three components: the large subunit (FRHA) that harbors the [NiFe] center and oxidizes H_2_, and the small subunit (FRHG), which contains three [4Fe–4S] clusters to transfer electrons to the flavin adenine dinucleotide (FAD) located in the F_420_‐reducing module (FRHB, harboring an additional [4Fe–4S] cluster). FRHABG dimerizes and forms dodecameric assemblies of about 16 nm in diameter with a spherical shape and a hollow core.^[^ ^2^ ^,^ ^4^ ^]^ The dodecameric assembly has been hypothesized to provide stability, protect redox centers, or keep the metal clusters at optimal distances.^[^ ^2^ ^,^ ^5^ ^]^

The structural description of the [NiFe]‐containing FRH from methanogenic archaea has been restricted to the terrestrial model organisms *Methanothermobacter marburgensis* and *Methanosarcina barkeri*.^[^ ^2^ ^,^ ^4^ ^,^ ^5^ ^]^ Here, by characterizing the native hydrogenase from the marine methanogen *Methanothermococcus thermolithotrophicus* ^[^ ^8^ ^]^ (belonging to the *Methanococcales* order), we provided an unprecedented functional understanding of this enzyme via a synergistic approach of biochemistry, X‐ray crystallography, and spectroscopy.

## 2 Results and Discussion

**Figure 1:**
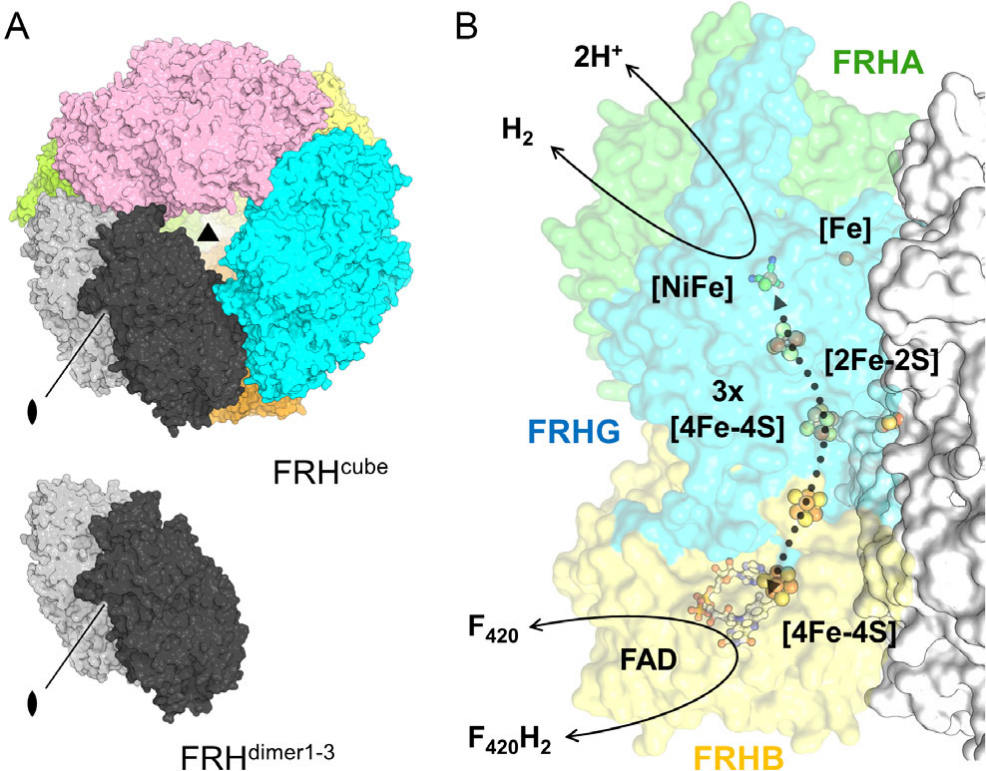
A) Overall architecture of the dodecameric and dimeric forms of [NiFe]‐containing *M* *t*FRH. Each FRH dimer is shown in a different color, with one of them being colored in two shades of gray to differentiate the two FRH protomers. B) Overall reaction scheme including the (metallo)cofactors for H_2_‐dependent F_420_ reduction. [Fe] and [NiFe] are part of FRHA, the 3x[4Fe–4S] and [2Fe–2S] clusters are part of FRHG, while FRHB harbors a [4Fe–4S] cluster and a FAD. Carbon, nitrogen, oxygen, sulfur, iron, and nickel are colored in green/white, blue, red, yellow, orange, and light green, respectively. The shown model is *Mt*FRH^dimer2^ with the [2Fe–2S] cluster copied from *Mt*FRH^dimer3^.

Despite its sequence conservation with the two other structural homologs (Figure S1–S3, Supporting Information), we found that the [NiFe]‐containing FRH from *M. thermolithotrophicus* (*Mt*FRH) exhibits a dynamic equilibrium between a dodecameric and dimeric state in solution, as highlighted by size exclusion chromatography (Figure S4A,B, Supporting Information) and native polyacrylamide gel electrophoresis (PAGE, Figure S4C,D, Supporting Information). It was therefore not surprising to observe several crystalline forms containing either a dodecameric cubic organization (*Mt*FRH^cube^) or only a single FRH dimer (*Mt*FRH^dimer1^ ^−3^, **Figure** **1**A).

Based on the four X‐ray crystallographic structures obtained (Table S1, Supporting Information), we confirmed that *Mt*FRH shares the basic organization previously observed^[^ ^2^ ^,^ ^4^ ^,^ ^5^ ^]^ with an elementary dimer of FRHABG heterotrimers that further builds into a dodecameric unit. Surface analyses through bioinformatics confirmed that the dodecameric state might be less stable in solution based on the structure of *M. barkeri* (*Mb*FRH, Figure S5, Supporting Information). However, considering the molecular crowding effect and the naturally high FRH expression (≈1% of total cytoplasmic protein),^[^ ^2^ ^]^ we propose that *Mt*FRH forms a dodecamer of ≈1.2 MDa in vivo. The different oligomers retain H_2_‐oxidase activity on native PAGE (Figure S4D, Supporting Information), and the purified enzyme has a specific activity of 186 ± 6 µmol of reduced F_420_ min^−1 ^mg^−1^ of *Mt*FRH at 50 °C with H_2_ as an electron donor. The electronic path is highly similar to structural homologs (Figure S6, Supporting Information), despite variations in the occupancy of the protomer‐bridging [2Fe–2S] cluster and FAD, which is likely an artifact of purification or oxidative damage, as the only reducing agent used during purification and crystallisation was dithiothreitol. FRH structures are superimposable without any movements between functional domains. The only noticeable difference is an N‐terminal extension in FRHG that forms a *β*‐hairpin, interacting with a *β*‐sheet of FRHA, which may enhance the thermal stability of the enzyme. Nevertheless, in the mesophilic *M. barkeri*, a similar *β*‐hairpin from FRHA also interacts with the same *β*‐sheet, potentially contributing to the overall stability of the protein (Figure S7, Supporting Information).

**Figure 2:**
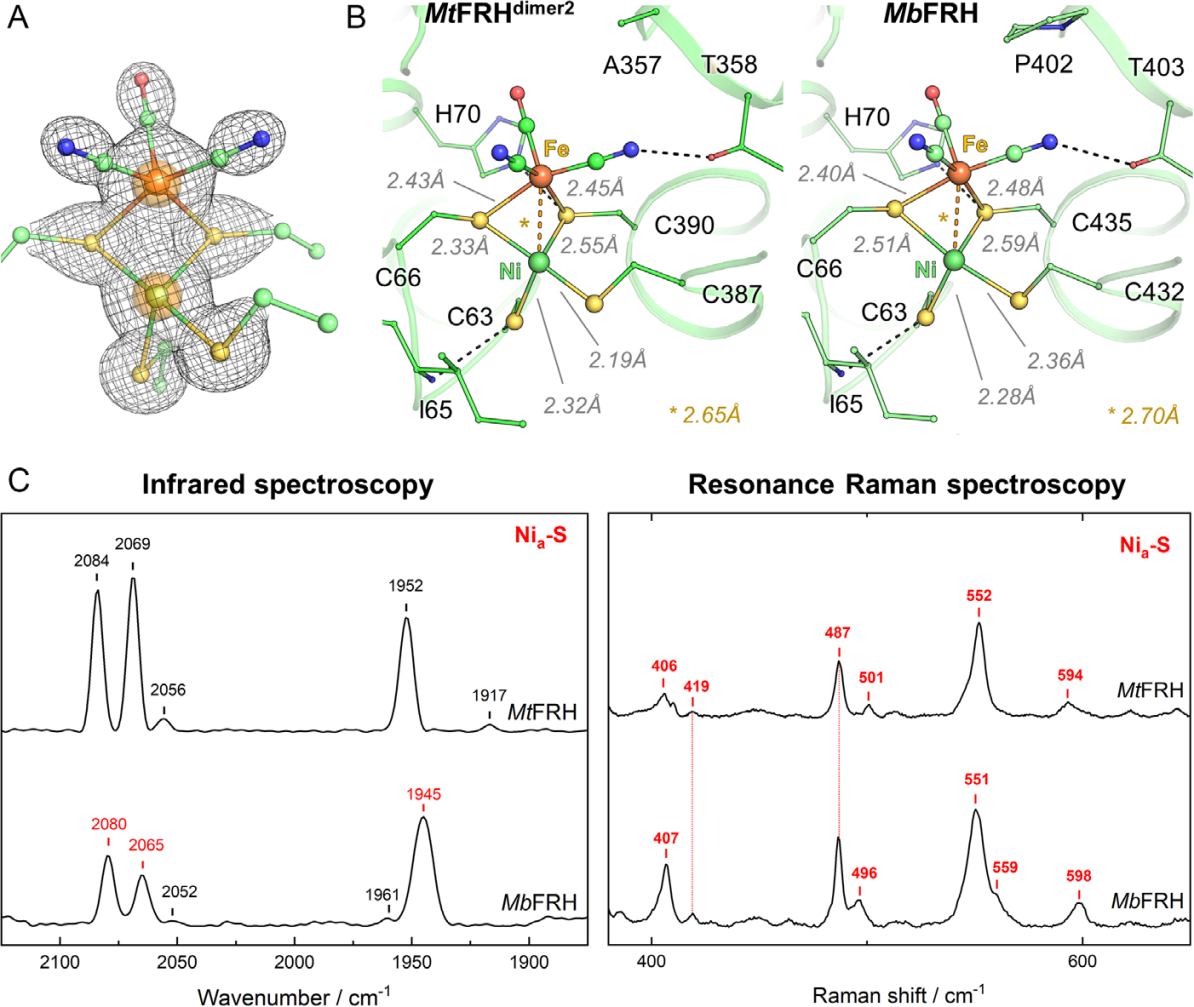
A) Close‐up of the [NiFe] catalytic site in *Mt*FRH^dimer2^. The 2*F*_o_‐*F*_c_ map of the metal and sulfur atoms is contoured to 3‐*σ*, and the 2*F*_o_‐*F*_c_ map of the nickel and iron is additionally contoured to 15‐*σ* and shown in a transparent orange surface. B) Active site and ligand‐metal distances comparison between *Mt*FRH and *Mb*FRH. Carbon, nitrogen, oxygen, sulfur, iron, and nickel are colored respectively in light green, blue, red, yellow, orange, and green. Dashed lines highlight close interaction between the [NiFe] center and the protein. C) Comparison of the IR and RR spectra of *Mt*FRH and *Mb*FRH crystals recorded at 80 K.^[^ ^4^ ^]^

Further depiction of the active site was done on the *Mt*FRH^dimer2^ form, as it exhibits the highest resolution of 1.65 Å. The deeply buried active site of *Mt*FRH^dimer2^ is similar to the structure described by Ilina et al.^[^ ^4^ ^]^ (Figure S8, Supporting Information), with a distorted seesaw geometry of the [NiFe] center and an apparent vacant site between the two metals (**Figure** **2**A).^[^ ^9^ ^,^ ^10^ ^]^ The ligand‐to‐metal distances are in the range of the *Mb*FRH structure, albeit with some shifts, particularly between the Cys66‐Ni and Cys387‐Ni (Figure 2B). However, the trans S–Ni–S angles (105° and 176°) and a short Ni–Fe distance of 2.65 Å, which are crucial for efficient hydrogen activation, are almost identical to those in *Mb*FRH (108°, 170°, and 2.70 Å intermetallic distance).^[^ ^4^ ^]^ Notably, we did not observe a partially occupied ordered water molecule in between the metals as modeled in the high‐resolution structure (1.45 Å) of the Ni_a_‐S state of Hyd‐1 from *E. coli* by Carr et al.^[^ ^11^ ^]^ This discrepancy might originate from the slightly better resolution or the presence of additional redox states with a bridging ligand in the Hyd‐1 structure, as indicated by an additional CN^−^ signal and overlapping CO bands in the corresponding infrared (IR) spectrum.^[^ ^11^ ^]^

To identify and further characterize the [NiFe] redox structural state, we performed spectroscopic studies by IR, resonance Raman (RR), and electron paramagnetic resonance (EPR). *In crystallo* spectroscopy on hydrogenases was recently established to verify the redox state of the active site in crystal structures^[^ ^4^ ^,^ ^11^ ^,^ ^12^ ^]^ and was applied to the *Mt*FRH^dimer3^ due to the abundance of crystalline material and the attainable crystal size.

RR spectroscopy on *Mt*FRH crystals confirmed the presence of oxidized [4Fe–4S] and [2Fe–2S] clusters (Figure S9, Supporting Information).^[^ ^13^ ^]^ The overall intensity of the Fe–S bands and observation of [2Fe–2S]^+^ specific signals indicate a more oxidized electron transfer (ET) chain in the as‐isolated state compared to *Mb*FRH.^[^ ^4^ ^]^

Moreover, IR and RR spectroscopy confirmed the presence of the Ni_a_‐S state (Table S2 and S3, Supporting Information), previously observed in *Mb*FRH.^4^ This state was postulated to be mandatory for the thermodynamically favorable binding of H_2_ and corroborated the vacant position observed in the crystal structure (Figure 2).^[^ ^4^ ^,^ ^14^ ^,^ ^15^ ^]^ IR spectra recorded at different temperatures and exposure to intense illumination did not significantly impact the spectroscopic pattern (Figure S10, Supporting Information), excluding temperature‐dependent shifts of the redox state equilibria or the presence of a light‐sensitive species, as observed recently in other hydrogenases.^[^ 16, 17, 18, 19 ^]^

In comparison to known and well‐characterized [NiFe]‐hydrogenases (Table S2, Supporting Information), the crystals turned out to be mostly redox‐inert (Figure S11, Supporting Information). The enrichment of the Ni_a_‐S state and its continued existence in the crystalline phase may have multiple reasons: e.g. i) The lack of the F_420_ substrate as terminal electron acceptor and the crystallisation temperature far from the theoretical optimal for the activity (20 °C versus 65 °C). ii) The ET chain may be partially disrupted as seen from a lower occupancy of the protomer‐bridging [2Fe–2S] cluster and FAD, impeding an efficient electron flow. iii) Overall, the redox states of the active site and the cofactors of the ET chain seem unusually decoupled since the former is partially reduced, while the latter are fully oxidized. This correlation was observed in both the crystalline (Figure 2C and S9, Supporting Information) as well as solution phase (Figure S12, Supporting Information). iv) Finally, the additional Fe site (Figure S6, Supporting Information), which was so far only observed for the FRH, is in ET distance to the active site and might have a stabilizing effect on the Ni_a_‐S state through redox cooperativity. Nevertheless, the comparison of Fe‐containing *Mt*FRH^dimer2^ and Fe‐depleted *Mt*FRH^cube^ does not reveal any structural rearrangement of the surrounding residues or the ones in the vicinity of the [NiFe] site. Therefore, the importance of the additional Fe site in modulating the redox properties of the [NiFe] center remains to be experimentally investigated.

**Figure 3:**
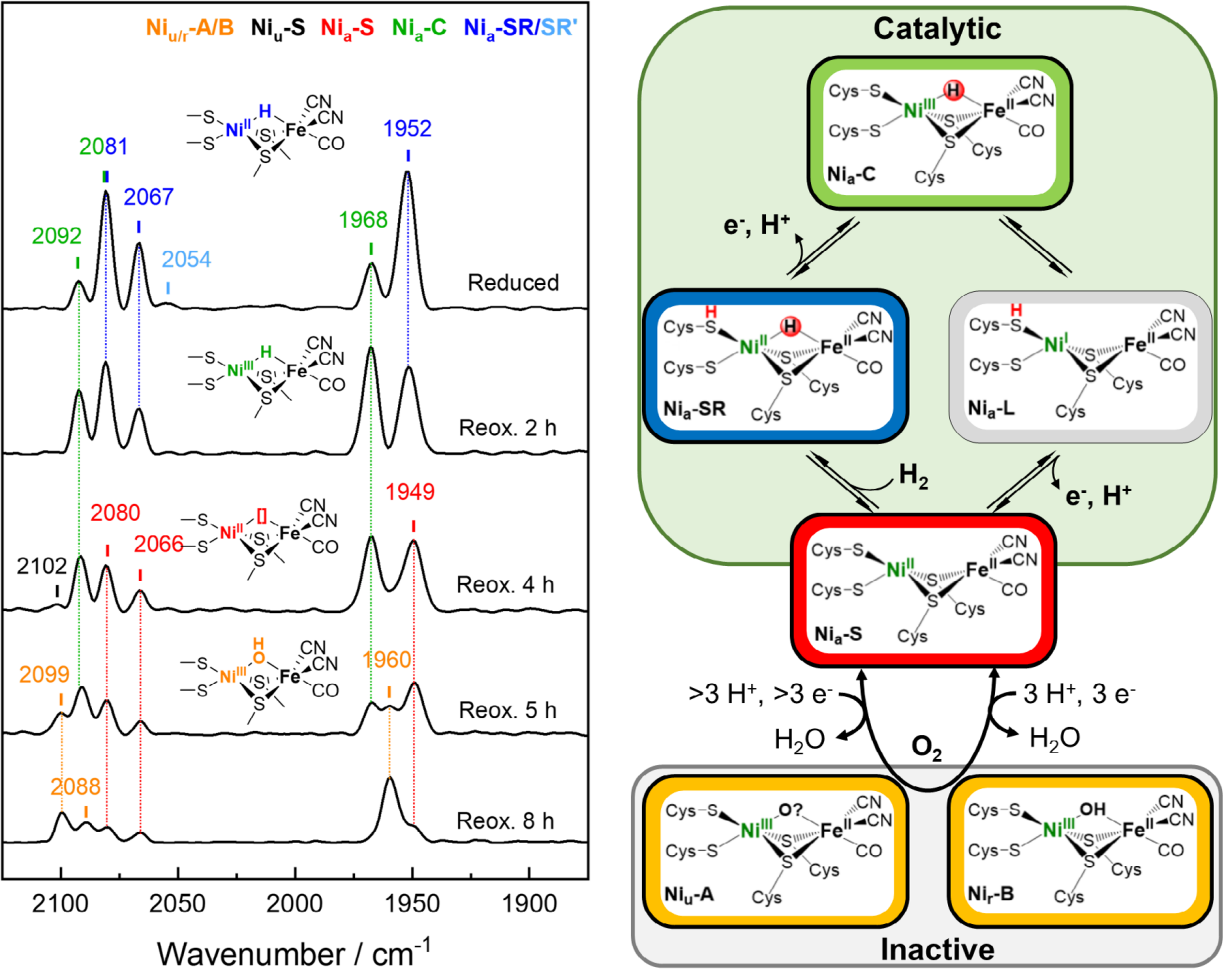
Monitoring the reoxidation by air of reduced *Mt*FRH in solution using IR spectroscopy at 283 K. The proposed catalytic mechanism and oxidative inactivation of the [NiFe] center are depicted on the right panel.^[^ ^1^ ^]^

We gained further insight into the redox chemistry of the [NiFe] center by performing IR spectroscopy on the protein in solution to probe different redox structural intermediates based on their characteristic CO and CN vibrations.^[^ ^20^ ^]^ After reduction with sodium dithionite, the active site resides mainly in the fully reduced Ni_a_‐SR and a minor population in the Ni_a_‐C state (**Figure** **3**). Slow diffusion of air into the IR cell gradually oxidizes the enzyme solution, forming the Ni_a_‐S state after 4 h. Further oxidation yields an IR signature, which could originate from the fully oxidized Ni_r_‐B and/or Ni_u_‐A state, which start to form after 5 h. These states were enriched even more after rapid oxidation with air of a sample initially reduced with pure H_2_ (Figure S12A, Supporting Information). Notably, as soon as the Ni_a_‐S state is formed, the overall intensity of the CO and CN signals starts to decrease drastically. After 8 h, only 40% of the initial intensity is present, indicating a degradation of the [NiFe] center. This may be due to a particular sensitivity of the unsaturated active site (Ni_a_‐S) to reactive oxygen species, which might be formed at the FAD during air reoxidation and/or an inefficient electron distribution throughout the two protomers, due to a partially impaired ET, see below. The soluble NAD^+^‐reducing [NiFe]‐hydrogenases (SH) from *Hydrogenophilus thermoluteolus* and *Cupriavidus necator*, both also classified as group 3, can resist such harmful conditions. While the former forms an unusual glutamate‐bound high‐valent redox state with sixfold nickel coordination in the presence of oxygen, the latter is oxygen‐tolerant, based on the reversible sulfoxygenation of the bridging cysteine residues.^[^ ^24^ ^]^

To verify the true nature of the most oxidized redox states and to further investigate the electronic structure of the other cofactors, EPR spectroscopy was applied. At 10 K, the as‐isolated sample remains almost EPR silent, indicating a fully oxidized iron–sulfur cluster chain and FAD (Figure S12B, Supporting Information) and a diamagnetic active site, in line with the observations from RR spectroscopy (Figure 2C and S9, Supporting Information). After exposure to H_2_, distinct signals related to the [4Fe–4S] cluster and a semiquinone radical can be observed. This verifies that, in solution, the enzymes can be reduced by H_2_ alone without the need for the substrate F_420_. Notably, no EPR signal could be detected for the reduced protomer‐bridging [2Fe–2S] cluster, in contrast to the related *Mb*FRH^4^, suggesting a potential interruption of the interprotomer ET. The signals from reduced [4Fe–4S] clusters and FAD disappear upon oxidation with air. Instead, weak features appear in the spectral range characteristic of paramagnetic redox states of the active site. Further analyses at 20 K revealed the typical rhombic signature of the Ni_u_‐A and the Ni_r_‐B states (Figure S12C, Supporting Information), indicating a rather conventional electronic structure of the most oxidized active site states as observed in standard hydrogenases.^[^ ^1^ ^]^ This is in contrast to most of the known [NiFe]‐hydrogenases from group 3, which form EPR silent species as most oxidized redox states under aerobic conditions, like the Ni_r_‐B‐like state in the SH from *Synechocystis* sp. PCC 6803 or *C. necator* or the oxygen‐resistant highly oxidized Ni_r_‐Hex state in the SH from *H. thermoluteolus* (see above).

Hydrogenases are sophisticated biocatalysts that combine high substrate selectivity, conferred by tunneling systems (Figure S13, Supporting Information), efficient turnover with rapid ET via FeS cluster relays, and active‐site stabilization by the protein matrix.^[^ ^10^ ^,^ ^25^ ^]^ While [NiFe] hydrogenases have been extensively studied, many aspects of their catalytic mechanism remain unresolved. In this study, we combine X‐ray crystallography with spectroscopic techniques to advance the identification and characterization of proposed catalytic intermediates in a group 3 [NiFe] hydrogenase. Among these intermediates, the presence of the hydrogen‐binding Ni_a_‐S state, first described by protein crystallography and complemented by *in crystallo* vibrational spectroscopy on *Mb*FRH^4^, has now been demonstrated in a thermophile marine homolog. Furthermore, the entire multitude of active‐site redox states was probed and assigned using complementary vibrational and EPR spectroscopy, marking a significant step toward a comprehensive mechanistic understanding of Group 3 [NiFe] hydrogenases.

## 3 Experimental Section

3.1

3.1.1

Cell growth, preparation of the cell extract, protein purification, characterization, and crystallisation were carried out under anoxic conditions, unless specified otherwise. All buffers were repeatedly degassed by vacuum and flushed with N_2_.

##### Strain and Cultivation

*M. thermolithotrophicus* DSM 2095 cells were obtained from the Leibniz Institute DSMZ‐German Collection of Microorganisms and Cell Cultures (Braunschweig) and cultivated in a previously described minimal medium with some modifications.^[^ ^26^ ^]^ Cells used for the purification of FRH^cube^ and FRH^dimer1^ were prepared as described in.^[^ ^27^ ^]^ FRH^dimer2^ and FRH^dimer^ ^3^ were prepared as in Jespersen et al.^[^ ^28^ ^]^ It is worth noting that the genome of *M. thermolithotrophicus* encodes two isoforms: a [NiFeSe]‐ and a [NiFe]‐containing enzyme. Besides the exchange of cysteine for selenocysteine that coordinates the active site in the [NiFeSe]‐FRH, the FRHA subunits between both enzymes show a highly conserved sequence amino acid identity, also when compared to the aforementioned structurally characterized homologs (Figure S1–3, Supporting Information ). To avoid sample contamination with the [NiFeSe]‐isoform for spectroscopic studies, *M. thermolithotrophicus* was cultivated at low Se concentrations, resulting in higher expression of the [NiFe]‐isoform.^[^ ^29^ ^]^

The medium contained, per liter: 558 mg KH_2_PO_4_ (final concentration 4.1 mM), 1 g KCl (13.4 mM), 25.13 g NaCl (430 mM), 840 mg NaHCO_3_ (10 mM), 368 mg CaCl_2_ · 2H_2_O (2.5 mM), 7.725 g MgCl_2_ · 6H_2_O (38 mM), 6.62 g (NH_4_)_2_SO_4_ (50.1 mM), 61.16 mg nitrilotriacetic acid (0.32 mM), 6.16 mg FeCl_2_·4H_2_O (0.031 mM), 0.38 mg Na_2_SeO_4_ (2 µM), 3.3 mg Na_2_WO_4_·2H_2_O (0.01 mM), and 2.42 mg Na_2_MoO_4_·2H_2_O (0.01 mM). The products were dissolved under constant stirring in a measuring cylinder with 750 ml of deionized H_2_O (dH_2_O). Resazurin was added to a final concentration of 0.0015 mM, and 10 ml of sulfur‐free trace elements (see below) were added subsequently. 50 mM 2‐morpholinoethanesulfonic acid (MES) was used as a buffer, and the pH was set to 6.2 with KOH. The media were filled up to a final volume of 1 L by the addition of deionized H_2_O.

The 100‐fold‐concentrated trace element solution was prepared by first dissolving 1.36 g nitrilotriacetic acid (7.1 mM) in 800 ml dH_2_O under magnetic stirring. The pH was adjusted to 6.6 by adding NaOH. Then, 89.06 mg MnCl_2_ · 4H_2_O (0.45 mM), 183.3 mg FeCl_3_ · 6H_2_O (0.68 mM), 60.27 mg CaCl_2_ · 2H_2_O (0.41 mM), 180.8 mg CoCl_2_ · 6H_2_O (0.76 mM), 90 mg ZnCl_2_ (0.66 mM), 37.64 mg CuCl_2_ (0.28 mM), 46 mg Na_2_MoO_4_·2H_2_O (0.19 mM), and 90 mg NiCl_2_ · 6H_2_O (0.38 mM) were added separately. The trace element mixture was filled to a final volume of 1 L with deionized H_2_O.

##### MtFRH Native Purification

All enzymes were purified using a similar protocol, the most optimized of which is detailed here. The frozen cells (55.3 g) were thawed under warm water and transferred to an anaerobic tent filled with an atmosphere of N_2_/CO_2_ (90:10%). Cells were lysed by osmotic shock through the addition of 220 ml lysis buffer (50 mM Tricine/NaOH pH 8.0, 2 mM dithiothreitol (DTT)). Cell lysate was homogenized by sonication: 11 cycles at 77% intensity with 30 pulses, followed by a 2 min break (probe MS76, SONOPULS Bandelin). Cell debris was removed anaerobically via centrifugation (21,000 g, 1 h at 10 °C). The supernatant was transferred to a Coy tent filled with N_2_/H_2_ atmosphere (97:3%) under yellow light at 20 °C and was filtered through a 0.2 µm filter. The filtered sample was separated into two halves, both applied to a 25‐ml DEAE fast‐flow column (Cytiva), which was previously equilibrated with lysis buffer. After loading, the column was washed with two column volumes (CV) of lysis buffer. A linear gradient of 0.1–0.6 M NaCl was applied for 120 min at a flow rate of 2.5 ml min^−1^ (12 CV) and fractions of 4 ml were collected. *Mt*FRH eluted between 0.3 and 0.373 M NaCl. The pooled fractions (≈100 ml) were diluted with 200 ml lysis buffer and filtered through a 0.2 µm filter. The filtered sample was separated into two halves, both loaded on a 15 ml Q Sepharose high‐performance column (GE Healthcare), washed with 2 CV of 0.15 M NaCl, and a gradient of 0.15–0.55 M NaCl was applied for 120 min with a flow rate of 1 ml min^−1^ (8 CV). Fractions of 1.6 ml were collected. *Mt*FRH eluted between 0.43 and 0.46 M NaCl. The pooled fractions were filtered and applied to a 10 ml hydroxyapatite type 1 (Bio‐Scale Mini CHT cartridges, BioRad) equilibrated with hydroxyapatite buffer (HAP buffer: 20 mM K_2_HPO_4_/HCl pH 7.0 and 2 mM DTT). The column was washed with 2 CV of HAP buffer, and the elution was performed with a gradient of 0.02–0.5 M K_2_HPO_4_ in 60 min at a flow rate of 2 ml min^−1^ (12 CV) with 3 ml fractions. *Mt*FRH eluted between 0.07 and 0.185 M K_2_HPO_4_, and the respective fractions were pooled. The pool was diluted 1:1 with hydrophobic interaction chromatography buffer (HIC buffer: 25 mM Tris/HCl pH 7.6, 2 M (NH_4_)_2_SO_4_ and 2 mM DTT) and filtered through a 0.2‐µm filter. The sample was applied onto a Source 15PHE 4.6/100 PE column (Cytiva) previously equilibrated with the HIC buffer. The column was washed with 2 CV of 25 mM Tris/HCl pH 7.6, 1.0 M (NH_4_)_2_SO_4_ and 2 mM DTT buffer. *Mt*FRH was eluted with a linear gradient of 1 to 0 M (NH_4_)_2_SO_4_ in 60 min at a flow rate of 0.7 ml min^−1^ and a fractionation volume of 1 ml. *Mt*FRH eluted between 0.65 and 0.5 M (NH_4_)_2_SO_4_, and the respective fractions were pooled. The pool was diluted with three volumes of lysis buffer, separated into two halves, and both were loaded on a Mono Q 5/50 GL (Cytiva). *Mt*FRH was eluted by applying a gradient of 0.1–0.5 M NaCl in 45 min at a flow rate of 1 ml min^−1^ and 0.5 ml fractions were collected. FRH eluted between 0.37 and 0.45 M NaCl, and these fractions were pooled and concentrated in a 100‐kDa‐cutoff centrifugation concentrator (6 ml, Merck Millipore) to a 300 µl final volume. Sample was separated in two halves, both applied onto a Superdex 200 Increase 10/300 GL (GE Healthcare), equilibrated in storage buffer (25 mM Tris/HCl pH 7.6, 10% (v/v) glycerol and 2 mM DTT) and the elution was performed at a flow rate of 0.4 ml min^−1^ and 0.4 ml fractions were collected. *Mt*FRH eluted in two Gaussian peaks at an elution volume of 10.4 and 11.4 ml, corresponding to pool A and pool B from Figure S4, Supporting Information. The fractions from the two peaks were pooled separately and concentrated by a 100‐kDa‐cutoff centrifugation concentrator. From the 55.3 g of *M. thermolithotrophicus* cells, a total of 1.64 mg FRH were purified. FRH was immediately used for crystallisation, the enzyme assays and for the native gels. The remaining sample was aliquoted and anaerobically flash frozen in liquid N_2_ and stored at −80 °C. *Mt*FRH lost its activity after more than one cycle of thawing and freezing.

##### Enzymatic Assay

Prior to the enzymatic assay, *Mt*FRH was incubated with 0.5 mM FAD for 30 min under an N_2_/H_2_ (97:3%) atmosphere. To remove FAD excess, the samples were concentrated in a 100‐kDa‐cutoff centrifugation concentrator, and the buffer was exchanged for fresh storage buffer. Activity measurements were performed with an Agilent Cary 60 UV–Vis spectrophotometer at 60 °C in 100 mM KH_2_PO_4_ buffer pH 7.5 in a 1 ml sealed quartz cuvette. The gas phase of the cuvette was exchanged several times with H_2_/CO_2_ (80:20%, 2 × 10^4^ Pa). To monitor the reduction of F_420_, 24.2 µM F_420_ was added to the buffer. The reaction was started by the addition of 0.33 µg *Mt*FRH. All experiments were performed in triplicate. For F_420_, a molar extinction coefficient of 41.50 mM^−1 ^cm^−1^ at 420 nm was experimentally determined in these conditions. The purification of F_420_ from *M. thermolithotrophicus* has been previously described.^[^ ^30^ ^]^

##### Protein Crystallisation

The purified enzymes were kept in storage buffer. Crystals were obtained anaerobically in a Coy tent filled with a N_2_/H_2_ (97:3%) atmosphere by initial screening at 20 °C using the sitting‐drop method on 96‐well MRC two‐drop crystallisation plates in polystyrene (SWISSCI) containing 90 µl of crystallisation solution in the reservoir or in CombiClover Jr. crystallisation plates (Molecular Dimensions). All enzymes were cocrystallised with the addition of 2 mM final FAD, except for *Mt*FRH^dimer^ ^3^, which crystallised as isolated. *Mt*FRH^cube^ and *Mt*FRH^dimer1^ were crystallised at a protein concentration of 16 mg ml^−1^. *Mt*FRH^dimer^ ^2^ and *Mt*FRH^dimer^ ^3^ were crystallised at a concentration of 6.1 and 38.8 mg ml^−1^, respectively. The crystallisation solutions in which the different crystals were obtained are the following: *Mt*FRH^cube^, 1.5 M LiSO_4_ and 100 mM HEPES pH 7.5; *Mt*FRH^dimer1^, 45% (w/v) pentaerythritol ethoxylate (3/4 EO/OH) 270, 100 mM HEPES pH 7.5, and 200 mM (NH_4_)_2_SO_4_; *Mt*FRH^dimer^ ^2^, 45% (w/v) pentaerythritol propoxylate (17/8 PO/OH) and 100 mM Tris pH 8.5; and *Mt*FRH^dimer^ ^3^, 30% (v/v) polyethylene glycol 400, 100 mM MES pH 6.5, and 200 mM LiSO_4_.

##### Data Collection, Processing, and Refinement

All crystals were directly frozen in liquid nitrogen, except for the crystal of *Mt*FRH^cube^ that was first soaked in the crystallisation solution supplemented with 30% (v/v) glycerol. Data were collected at 100 K at the different synchrotrons and beamlines stated in Table S1, Supporting Information. All data were integrated with *autoPROC*,^[^ ^31^ ^]^ except for *Mt*FRH^cube^ and *Mt*FRH^dimer1^, which were processed with XDS and scaled with scala from the CCP4 suite. All models were solved by molecular replacement using Phaser from *PHENIX*. FRH from *M. marburgensis* (PDB code 4OMF) was used as a template to solve *Mt*FRH^dimer1^. All other structures were solved by using *Mt*FRH^dimer1^ as a template for molecular replacement. All models were manually optimized with *COOT*.^[^ ^32^ ^]^ Refinement was performed with *phenix.refine* ^[^ ^33^ ^]^ or *BUSTER* ^[^ ^34^ ^]^ without applying noncrystallography symmetry for the models refined with *PHENIX*. All models were refined by using a translation‐libration screw and by adding hydrogens in riding positions. The different structures were validated by the MolProbity^[^ ^35^ ^]^ tool integrated with *PHENIX*. Only *Mt*FRH^dimer2^ was deposited in the Protein Data Bank with hydrogens on the protein.

##### Native Gels

High‐resolution clear native PAGE (hrCN PAGE) was prepared and run as in Lemaire et al.^[^ ^36^ ^]^ The whole process was performed anaerobically in an anoxic chamber N_2_/CO_2_ (90:10%). Glycerol (20% v/v final) was added to each sample, and 0.001% (w/v) Ponceau S served as a marker for protein migration. The electrophoresis cathode buffer contained 50 mM Tricine, 15 mM Bis‐Tris, pH 7, 0.05% (w/v) sodium deoxycholate, 0.01% (w/v) dodecyl maltoside, and 2 mM DTT. The anode buffer contained 50 mM Bis‐Tris buffer pH 7 and 2 mM DTT. hrCN PAGE was carried out using a 5–15% linear polyacrylamide gradient and gels were run with a constant 40 mA current (PowerPac Basic Power Supply, Bio‐Rad). After electrophoresis, protein bands were visualized by viologen‐based hydrogenase activity. In‐gel activity staining was performed in 10 mL of anoxic 25 mM Tris/HCl buffer, pH 7.6, 2 mM DTT, 5 mM methylviologen, and 1 mM of 2,3,5‐triphenyltetrazolium chloride. The latter component was used to maintain the staining under oxygen exposure. After electrophoresis, the native gels and the reaction mixture were anaerobically transferred to 1 L Duran Bottles, and the gas phase was exchanged for 100% N_2_ at 50 kPa. The reaction was started by the addition of 100% H_2_. The reaction was performed at 50 °C and stopped by opening the bottle under a fume hood, thereby exposing the protein to oxygen.

##### Figure Preparation

All figures presenting a structural model were generated with PyMOL version 2.2.0. Figure S1–3, Supporting Information, were generated with ESPript^[^ ^37^ ^]^ by using a sequence alignment from *MUSCLE* ^[^ ^38^ ^]^ and *Mt*FRH^dimer1^ as a structural template for the secondary structure. All figures that display spectroscopic data were created using Origin 2025.

##### Sample Preparation for Spectroscopic Experiments

Protein solution (0.1 mM) of *Mt*FRH in the as‐isolated state was measured by IR and EPR spectroscopy. For H_2_ reduction, the protein solution was purged with humidified 100% H_2_ in an anaerobic tent (N_2_/H_2_, 97:3%) for 30 min, while reduction with sodium dithionite (20‐fold excess) was done in an anaerobic glovebox (100% N_2_). The fast reoxidation of the protein solution was conducted using humidified air. Protein crystals without further treatment (as isolated), after soaking in K_3_[Fe(CN)_6_] or sodium dithionite for 5 min, and washing in crystallisation solution, were transferred to a MgF_2_ plate for IR and RR spectroscopic experiments and frozen in liquid nitrogen.

##### IR Spectroscopy

Protein solution (0.1 mM) of *Mt*FRH was transferred into a homemade, gastight IR transmission cell containing two CaF_2_ windows separated by a 50‐μm Teflon spacer. The sample chamber was purged with dry air, which allowed for monitoring the reoxidation of the sample (Figure 3) due to the slow diffusion of oxygen into the transmission cell. All IR spectra were recorded with a spectral resolution of 2 cm^−1^ on a Bruker Tensor 27 FTIR spectrometer using a liquid‐N_2_ cooled MCT detector. Absorbance spectra were calculated using the buffer single channel spectrum as reference. The Bruker OPUS software 6.5 or higher was used for data evaluation.

##### IR Microscope

IR spectra of *Mt*FRH protein crystals were recorded using a Bruker Tensor 27 FTIR spectrometer connected to a Bruker Hyperion 3000 IR microscope equipped with a 20× IR transmission objective and an MCT detector. A liquid‐N_2_‐cooled cryo‐stage (Linkam Scientific Instruments) was used for temperature control.

##### EPR Spectroscopy

A 9.3‐GHz X‐Band continuous‐wave Bruker EMXplus spectrometer equipped with an ER 4122 SHQE resonator was used for recording EPR spectra. The parameters were set as follows: modulation amplitude: 10 G, modulation frequency: 100 kHz, and microwave power: 1 mW. An Oxford EPR 900 He‐flow cryostat equipped with an Oxford ITC4 controller was used to control the temperature. All data were processed using the Bruker Xenon software (version 1.1b58). Simulation of the EPR spectra was done using the MATLAB toolbox EasySpin version 5.2.36.^[^ ^39^ ^]^

##### RR Spectroscopy

RR spectra were recorded using a LabRam HR‐800 Jobin Yvon confocal Raman microscope spectrometer connected to a liquid‐N_2_‐cooled charge‐coupled device. The spectra were accumulated at 80 K using a liquid‐N_2_‐cooled cryo‐stage (Linkam Scientific instruments). The 458 line of an Ar^+^ ion laser at 1 mW or the 568‐nm line of a Kr^+^ ion laser at 2 mW was used for excitation. The laser beam was focused at a 2–4 μm spot on the surface of a single crystal. The RR spectra displayed consist of an average of 30–40 individual spectra, each accumulated for 100–180 s. Frequency calibration was performed using toluene as an external and the band of phenylalanine at 1004 cm^−1^ as an internal standard.

## Conflict of Interest

The authors declare no conflict of interest.

## Author Contributions

**Marion Jespersen** and **Christian Lorent** contributed equally to this work. **Marion Jespersen**: conceptualization, data curation, formal analysis, investigation, methodology, validation, visualization, writing—original draft, writing—review and editing; **Christian Lorent**: investigation, formal analysis, validation, methodology, writing—review and editing; **Olivier N. Lemaire**: investigation, methodology, validation, writing—review and editing; **Ingo Zebger**: conceptualization, funding acquisition, investigation, supervision, validation, writing—review and editing; **Tristan Wagner**: conceptualization, data curation, funding acquisition, formal analysis, investigation, methodology, supervision, validation, writing—review and editing.

## Supporting information

Supplementary Material

## Data Availability

The data that support the findings of this study are openly available in [PDBe, Protein Data Bank] at [https://doi.org/10.2210/pdb9r6z/pdb; https://doi.org/10.2210/pdb9r51/pdb; https://doi.org/10.2210/pdb9r52/pdb; https://doi.org/10.2210/pdb9r5i/pdb].
