# Supplementary material for: Structural and Spectroscopic Insights into Catalytic Intermediates of a [NiFe]‐hydrogenase from Group 3
Source: Chembiochem. 2025 Oct 13;26(21):e202500692. doi: 10.1002/cbic.202500692 (PMC12596923; doi:10.1002/cbic.202500692)
Supplement: Supplementary file 1 — Supplementary Material [file CBIC-26-e202500692-s001.pdf]

Structural and spectroscopic insights into catalytic intermediates of a [NiFe]-hydrogenase from group 3

Marion Jespersen, Christian Lorent, Olivier N. Lemaire, Ingo Zebger and Tristan Wagner

Table of contents:

Tables S1-4

Figures S1-S13

References

Table S1. X-ray analysis statistics for *Mt*FRH.

	FRH^{cube}	FRH^{dimer1}	FRH^{dimer2}	FRH^{dimer3}
**Data collection**				
Synchrotron source, beamline	ESRF, BM30A–FIP	ESRF, BM30A–FIP	SLS, X06SA	SOLEIL, Proxima I
Wavelength (Å)	1.38710	1.38710	1.00003	1.73891
Space group	$P2_13$	$P3_221$	$C222_1$	$P3_121$
Resolution (Å)	49.10 – 2.85 (3.00 – 2.85)	48.86 – 2.30 (2.42 – 2.30)	69.27 – 1.65 (1.79– 1.65)	127.41 – 3.13 (3.35– 3.13)
Cell dimensions				
a, b, c (Å)	282.07, 282.07, 282.07	192.71, 192.71, 386.21	95.03, 163.48, 128.83	196.61, 196.61, 192.07
α, β, γ (°)	90, 90, 90	90, 90, 120	90, 90, 90	90, 90, 120
R_{merge} (%)[a]	21.7 (149.7)	9.1 (59.7)	11.2 (235.4)	30.4 (244.9)
R_{pim} (%)[a]	8.5 (57.2)	4.2 (30.4)	2.6 (56.0)	6.9 (54.5)
$CC_{1/2}$[a]	0.988 (0.433)	0.998 (0.565)	0.999 (0.588)	0.997 (0.620)
I/σ_I[a]	6.0 (1.2)	13.6 (2.8)	16.1 (1.5)	10.0 (1.6)
Spherical completeness[a]	100.0 (100.0)	99.8 (99.1)	77.3 (18.2)	69.0 (18.8)
Ellipsoidal completeness[a]	/	/	94.1 (59.9)	95.1 (67.7)
Redundancy[a]	7.6 (7.8)	5.7 (4.7)	20.1 (18.4)	20.2 (21.1)
Nr. unique reflections[a]	173,025 (25,112)	365,266 (52,491)	92,187 (4,460)	52,281 (2,616)
**Refinement**				
Resolution (Å)	31.34 – 2.85	31.55 – 2.30	64.41 – 1.65	77.83 – 3.13
Number of reflections	172,727	365,025	92,177	52,264
R_{work}/R_{free}[b] (%)	18.23/21.01	17.98/20.74	14.11/18.26	17.44/20.59
Molecules/asymmetric unit	4 x (FRHABG)	4 x (FRHABG)	1 x (FRHABG)	2 x (FRHABG)
Number of atoms				
Protein	28,194	28,200	14,163[d]	14,094
Ligands/ions	562	735	235	389
Solvent	70	1888	725	0
Mean B-value ($Å^2$)	82.00	54.94	36.11	82.44
Molprobity clash score, all atoms	4.46	3.32	1.32	2.31
Ramachandran plot				
Favoured regions (%)	95.93	96.42	96.67	95.10
Outlier regions (%)	0.19	0.22	0.22	0.17
rmsd[c] bond lengths (Å)	0.006	0.007	0.008	0.006
rmsd[c] bond angles (°)	0.878	1.005	1.189	0.935
PDB code	9R6Z	9R51	9R52	9R5I

[a] Values relative to the highest resolution shell are within parentheses. [b] R_{free} was calculated as the R_{work} for 5 % of the reflections that were not included in the refinement. [c] rmsd, root mean square deviation. [d] The protein backbone contains hydrogen.

Table S2. Characteristic CO and CN stretching bands of the redox states of *Mt*FRH in comparison to data from other group 3 [NiFe]-hydrogenases. SH is the abbreviation for soluble hydrogenase.

Redox State	Organism / Enzyme	ν(CO) / cm^{-1}	$ν(CN)_{as}$ / cm^{-1}	$ν(CN)_s$ / cm^{-1}	Reference
Ni_u-A	*M. thermolithotrophicus* FRH	**1960/2**	**2088**	**2099**	This work
	A. vinosum SH	1962[a]	2086[a]	-	Long et al.[1]
Ni_r-B	*M. thermolithotrophicus* FRH	**1960/2**	**2088**	**2099**	This work
	A. vinosum SH	1962[a]	2086[a]	-	Long et al.[1]
Ni_r-B-like[b]	*Synechocystis sp. PCC 6803*	1957	2076	2088	Germer et al.[2]
	C. necator SH	1957	2080	2090	Horch et al.[3]
$Ni(III)_r$-Hex	*H. thermoluteolus* SH	1964	2087	2098	Preissler et al.[4]<br>Kulka-Peschke et al.[5]
Ni_u-S	*M. thermolithotrophicus* FRH	**1954**[c]	**2090**[c]	**2102**[c]	This work
Ni_a-S	*M. thermolithotrophicus* FRH	**1949**	**2066**	**2080**	This work
	M. barkeri FRH	1945[d]	2065[d]	2080[d]	Ilina et al.[6]
	P. furiosus SH1	1947	-	-	Greene et al.[7]
	Synechocystis sp. PCC 6803	1947	2078	2093	Germer et al.[2]
	C. necator SH	1946	2080	2090	Horch et al.[3]
	H. thermoluteolus SH	1951	2076	2089	Preissler et al.[4]<br>Kulka-Peschke et al.[5]
Ni_a-C	*M. thermolithotrophicus* FRH	**1968**	**2081**	**2092**	This work
	P. furiosus SH1	1967	-	-	Greene et al.[7]
	Synechocystis sp. PCC 6803	1968	2079	2093	Germer et al.[2]
	C. necator SH	1959	2081	2091	Horch et al.[3]
	H. thermoluteolus SH	1971	2076	2089	Preissler et al.[4]<br>Kulka-Peschke et al.[5]
	A. vinosum SH	1969[e]	-	2093	Long et al.[1]
Ni_a-SR	*M. thermolithotrophicus* FRH	**1952**	**2067**	**2081**	This work
	P. furiosus SH1	1953	-	-	Greene et al.[7]
	Synechocystis sp. PCC 6803	1955	2063	2079	Germer et al.[2]
	C. necator SH	1946	2081	2091	Horch et al.[3]
	H. thermoluteolus SH	1958	2062	2076	Preissler et al.[4]<br>Kulka-Peschke et al.[5]
	A. vinosum SH	1947	-	-	Long et al.[1]
Ni_a-SR'	*M. thermolithotrophicus* FRH	**1939**	**2054**	-	This work
	P. furiosus SH1	1939	-	-	Greene et al.[7]
	C. necator SH	1922	2052	2070	Horch et al.[3]
	H. thermoluteolus SH	1943	2048	2062	Preissler et al.[4]<br>Kulka-Peschke et al.[5]

[a] Assigned based on corresponding EPR spectra in Long et al.[1], [b] EPR silent, [c] assigned based on similar frequencies observed for the same mode of the Ni_u-S state in standard [NiFe]-hydrogenases and the enrichment of this state during aerobic reoxidation[8], [d] recorded at 80 K, [e] assigned as Ni_a-S in Long et al.[1]

Table S3. Characteristic Fe-CO/CN bands of the Ni_a-S state of *Mt*FRH in comparison to data from other [NiFe]-hydrogenases. MBH and RH stand for membrane-bound hydrogenase and regulatory hydrogenase, respectively. The main band is highlighted in bold.

Organism / Enzyme	Fe-CN/CO / cm^{-1}	Reference
M. thermolithotrophicus FRH	406, 419, 487, 501, **552**, 594	This work
M. barkeri FRH	407, 419, 487, 495, **551**, 559, 598	Ilina et al.[6]
C. necator RH	410, 417, 490, 495, **553**, 563, 600	Horch et al.[9]
C. necator MBH	426, 448, 490, 504, **552**, 564, 590	Siebert et al.[10]

Table S4. Characteristic g-values of the Ni_u-A and Ni_r-B states of *Mt*FRH in comparison to data from other [NiFe]-hydrogenases. MBH and SH stand for membrane-bound hydrogenase and soluble hydrogenase, respectively.

Redox state	Organism / Enzyme	g_1	g_2	g_3	Reference
Ni_u-A	*M. thermolithotrophicus* FRH	2.30	2.25	2.01	This work
	D. v. miyazaki F MBH	2.32	2.24	2.01	Albracht[11]
	D. gigas MBH	2.31	2.23	2.02	Teixeira et al.[12]
	A. vinosum SH	2.30	2.23	2.02	Long et al.[1]
Ni_r-B	*M. thermolithotrophicus* FRH	2.31	2.16	2.00	This work
	D. v. miyazaki F MBH	2.33	2.16	2.01	Albracht[11]
	D. gigas MBH	2.33	2.16	2.02	Teixeira et al.[12]
	A. vinosum SH	2.37	2.16	2.02	Long et al.[1]

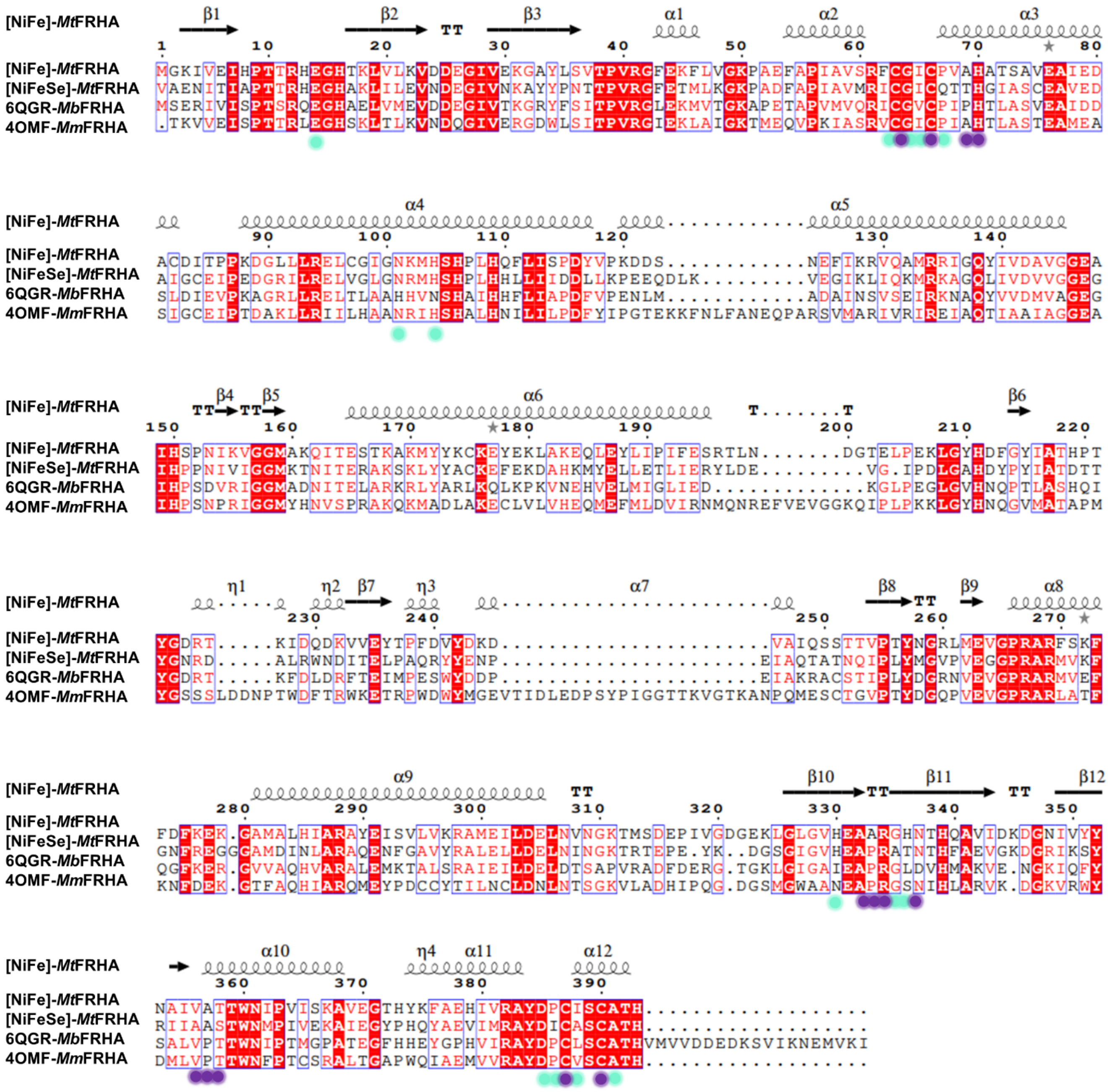

Figure S1. Alignment of FRHA from the [NiFe]-*Mt*FRH (WP_018154259), [NiFeSe]-*Mt*FRH (WP_245547917, Cys386 in this sequence is a SeCys), *Mb*FRH (PDB 6QGR) and *Mm*FRH (PDB 4OMF). Sequence alignment was done using MUSCLE, and the picture was created with ESPript 3.0.[13] Residues part of the first and second [NiFe] shell of interactions are highlighted by purple and cyan dots, respectively.

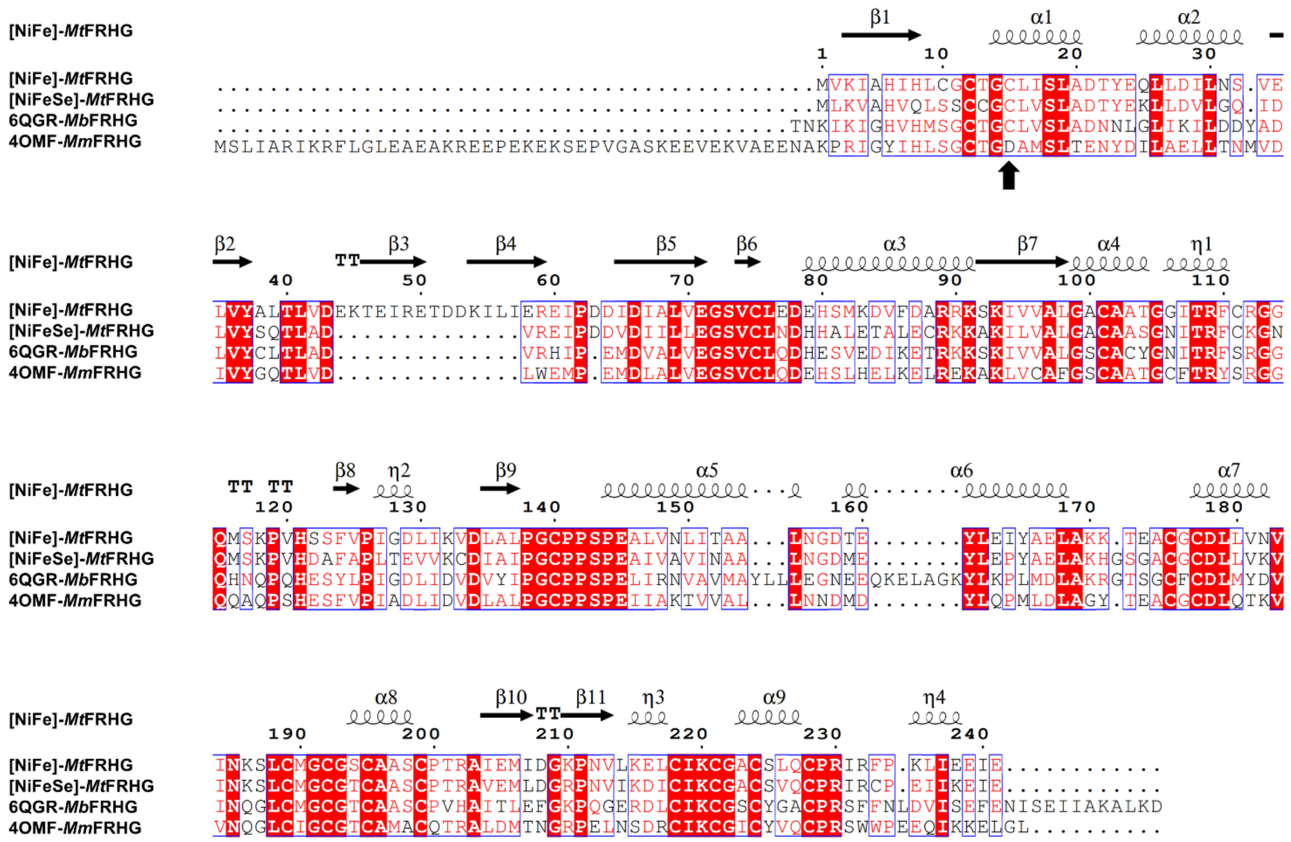

Figure S2. Alignment of FRHG from the [NiFe]-*Mt*FRH (WP_018154257), [NiFeSe]-*Mt*FRH (WP_018154657), *Mb*FRH (PDB 6QGR) and *Mm*FRH (PDB 4OMF). Sequence alignment was done using MUSCLE, and the picture was created with ESPript 3.0.[13] In *Mm*FRHG, the proximal [4Fe-4S] cluster is coordinated by Asp60 (highlighted by a black arrow) compared to a canonical Cys.

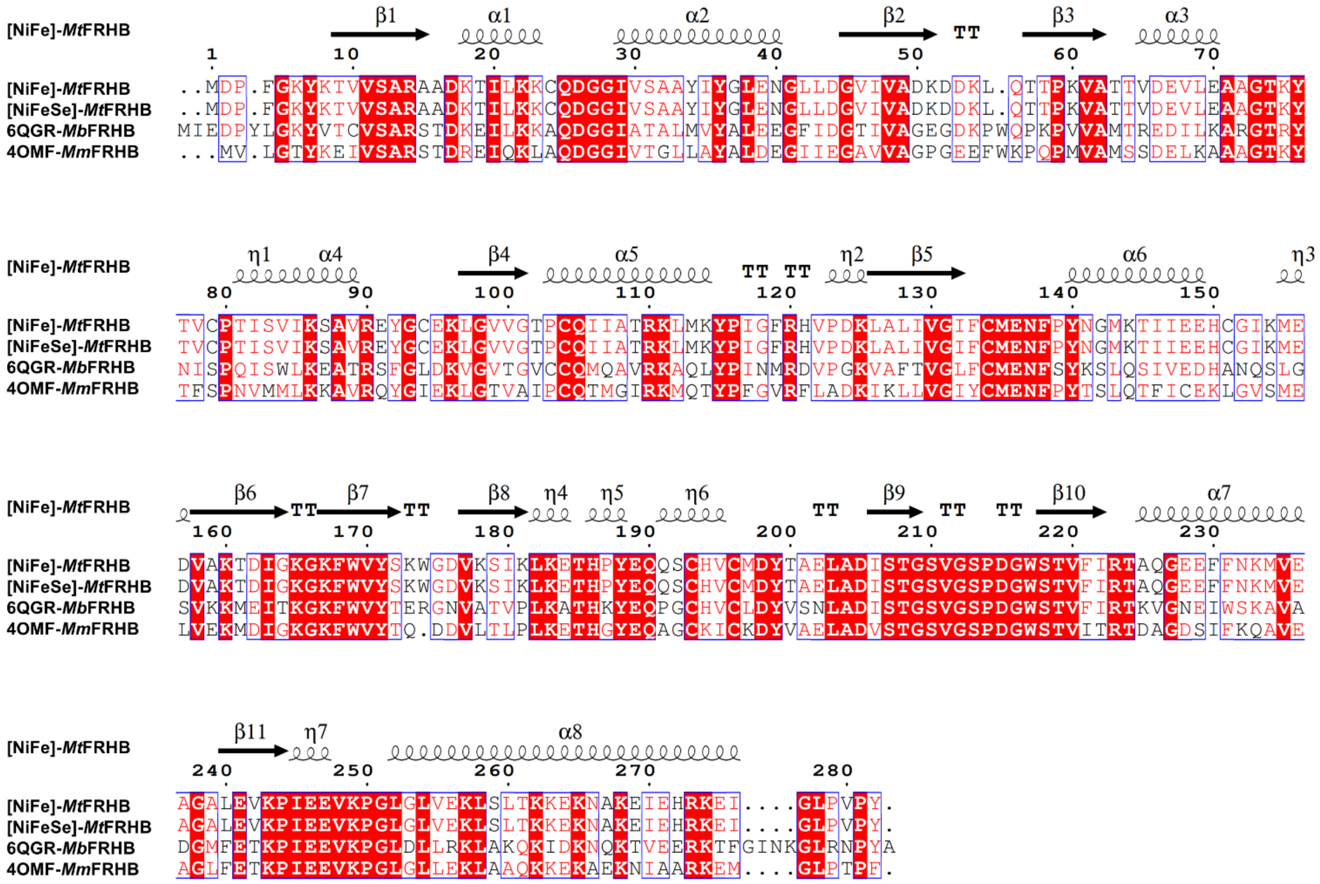

Figure S3. Alignment of FRHB from the [NiFe]-*Mt*FRH and [NiFeSe]-*Mt*FRH (WP_018153424), *Mb*FRH (PDB 6QGR) and *Mm*FRH (PDB 4OMF). Sequence alignment was done using MUSCLE, and the picture was created with ESPript 3.0.[13] The two isoforms of *Mt*FRHB share the same nucleotide sequence.

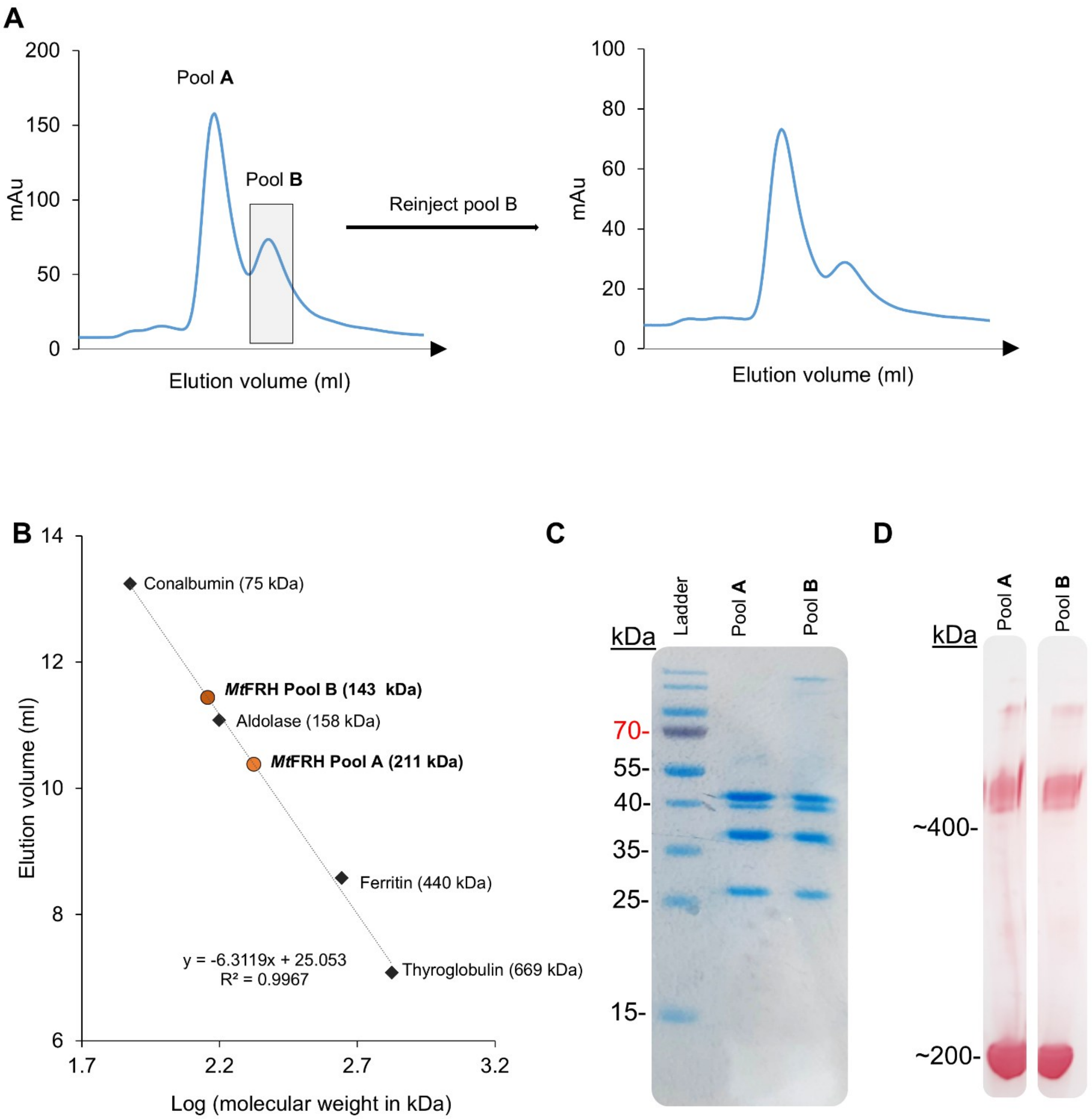

Figure S4. Dynamic oligomeric behaviour of [NiFe]-*Mt*FRH. A) Purified *Mt*FRH sample elutes in two major populations on a size exclusion chromatography, labelled as Pool A and Pool B. Reinjecting one population, here Pool B, results in the same profile (right chromatogram). *Mt*FRH eluted at an elution volume of 10.4 ml and 11.4 ml, corresponding to pool A and pool B, respectively. (B) The estimated molecular weights of the A and B populations correspond to sizes of 143 and 211 kDa. (C) SDS-PAGE profile from the two purified *Mt*FRH pools (5 µg per pool was loaded). (D) hrCN-PAGE of the two *Mt*FRH pools (5 µg loaded). The red staining is due to viologen-based triphenyl tetrazolium reduction upon H_2 oxidation by FRH.

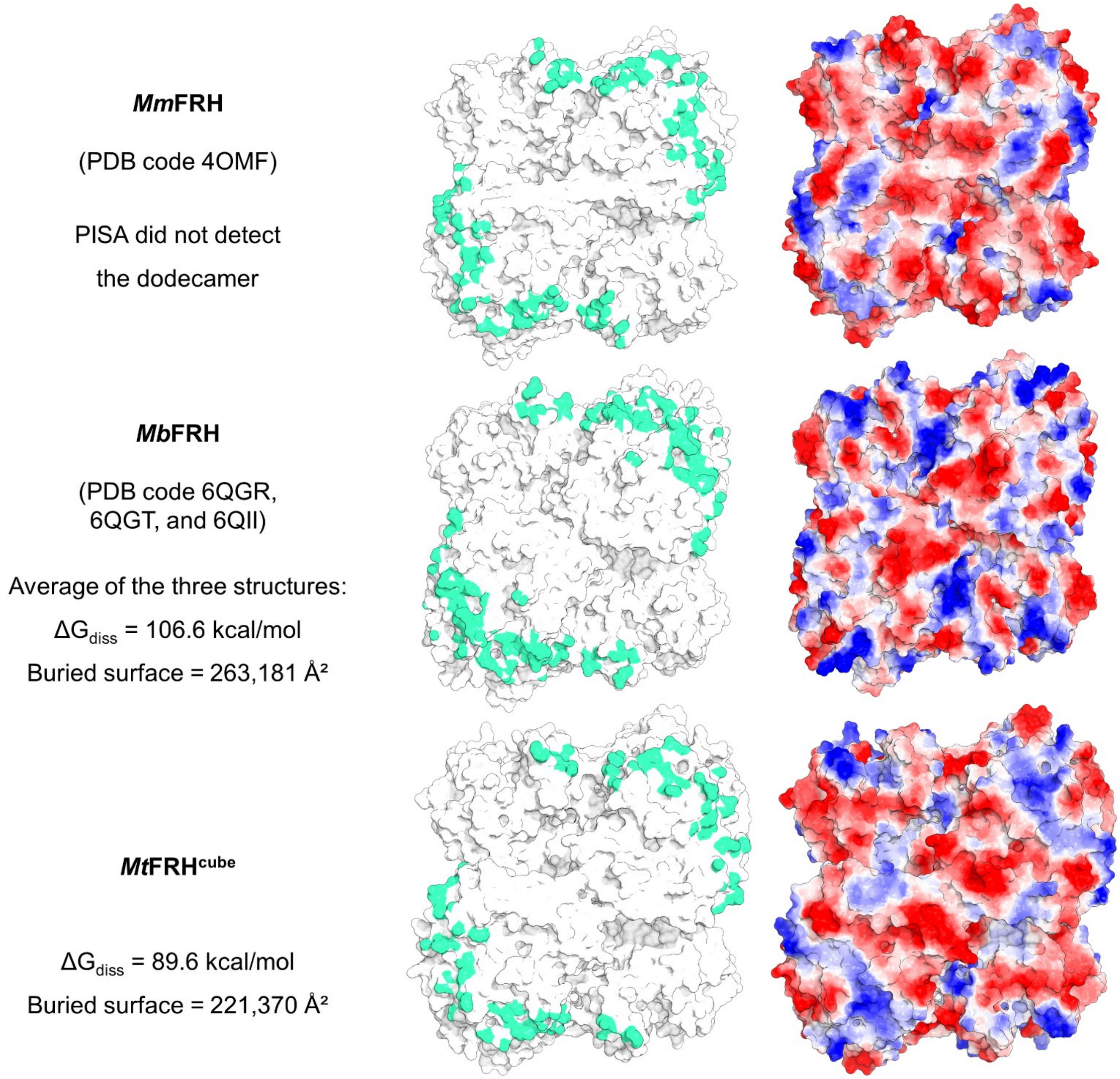

Figure S5. Dimer-dimer interaction in the dodecameric quaternary assembly. All models are represented as a surface. The cyan colour on the left panels highlights residues interacting with the surrounding FRH dimers in the dodecameric assembly, therefore pointing out the contacts involved in the dodecameric organisation. The blue and red colours on the right panel represent the electrostatic charges, ranging from negative (red) to positive (blue). The view is taken from inside the cube's hollow core in both panels.

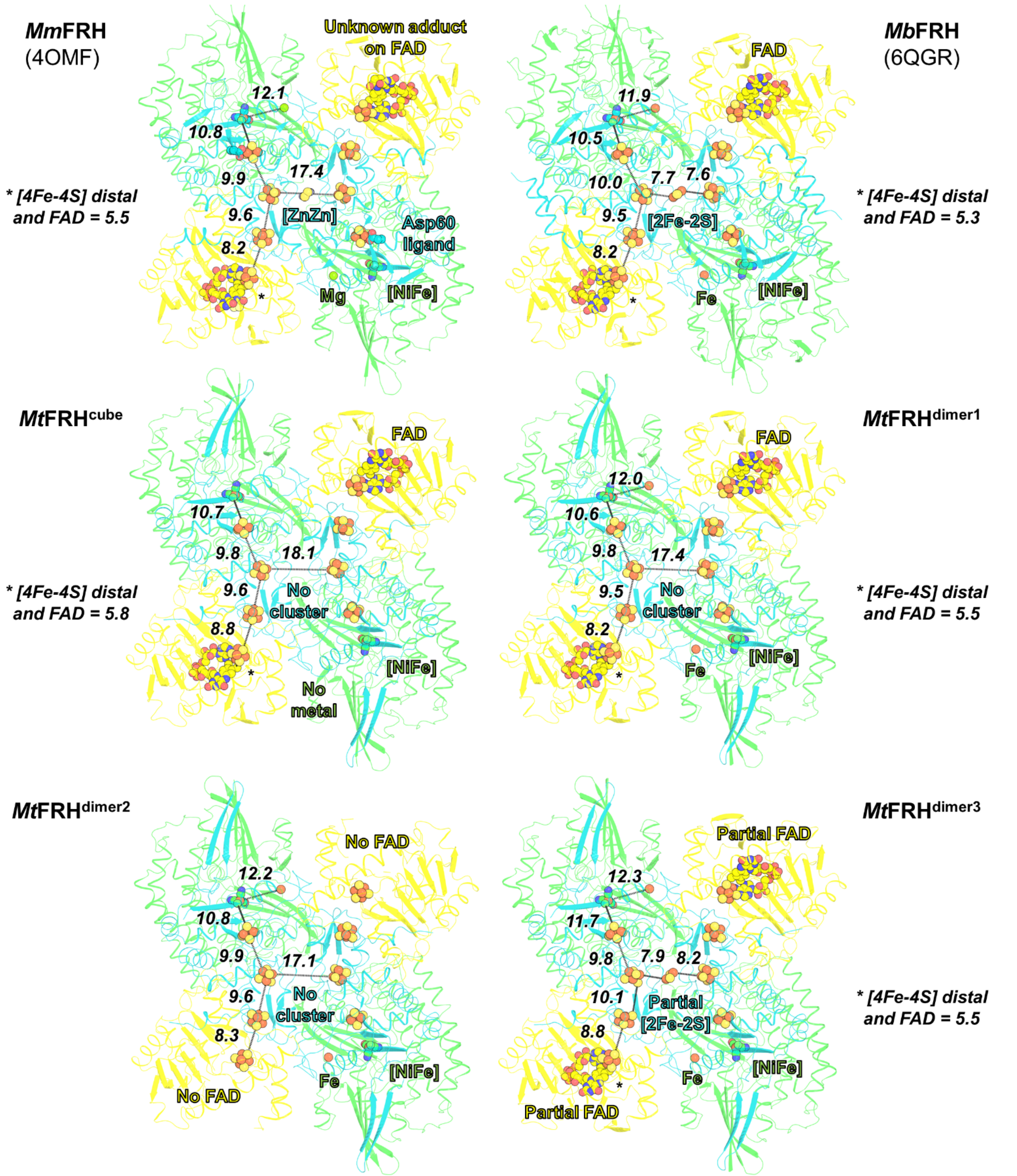

Figure S6. Overall structural comparison between the FRH structures. The electron transfer path, highlighted by dashed lines with the distances in Å, is shown. The differences in metallocofactors/FAD/[FeS]-cluster ligand composition for each form are summarised directly in the figure. For all models, FRHA, FRHG, and FRHB are coloured in green, cyan, and yellow, respectively. The metalloclusters, FAD, and Asp60 from *Mm*FRH are represented in balls and sticks. The carbon atoms of the ligands are coloured according to the respective subunit. Nitrogen, oxygen, sulfur, phosphorus, magnesium, zinc, iron, and nickel atoms are coloured blue, red, yellow, brown, chartreuse, grey, orange, and light green, respectively.

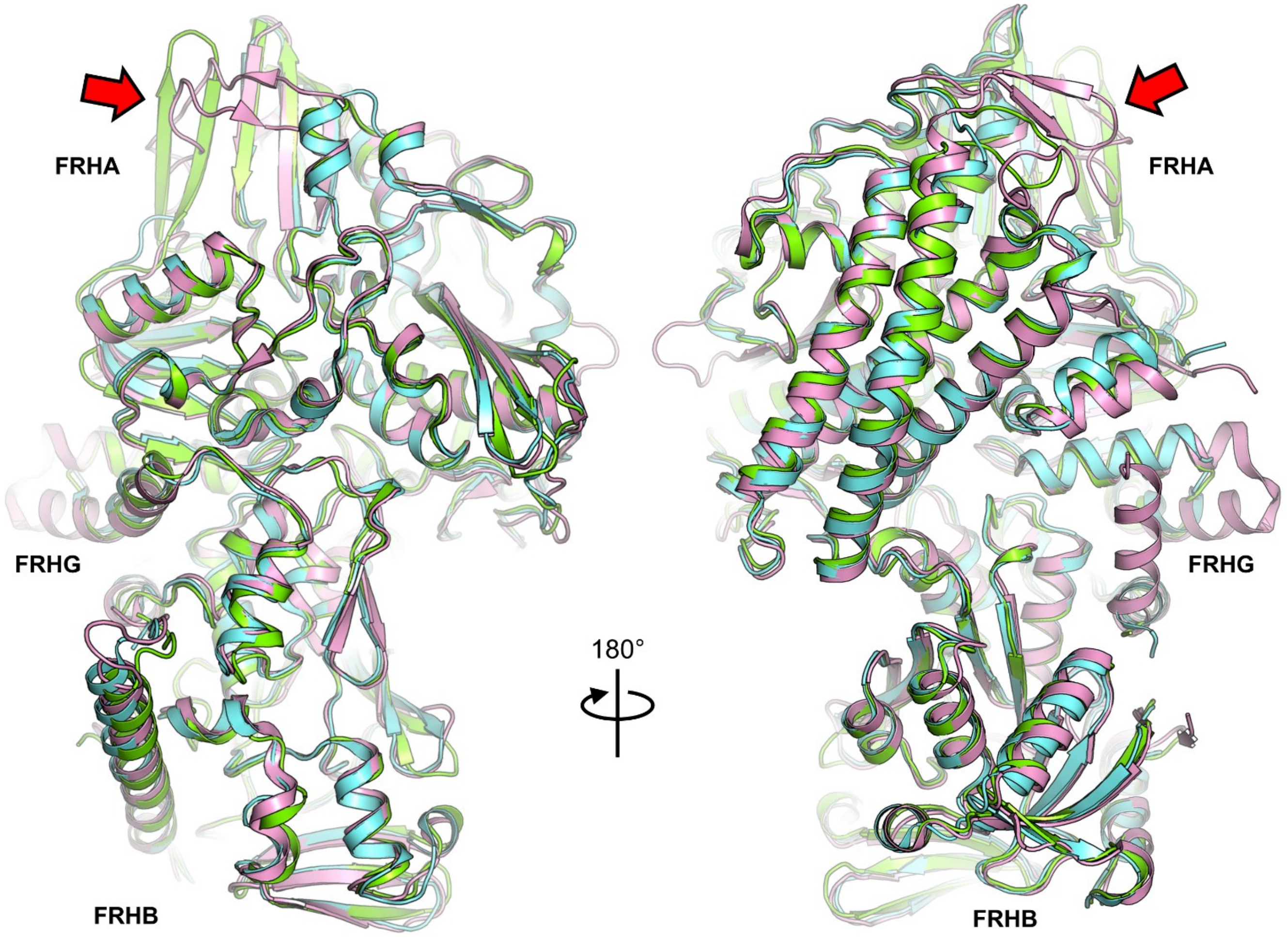

Figure S7. Superposition of all characterised FRH. The models are displayed in cartoons with *Mt*FRH^{dimer1}, *Mb*FRH (PDB 6QGR) and *Mm*FRH (PDB 4OMF) coloured green, pink and cyan, respectively. The extensions from *Mt*FRHG and *Mb*FRHA gathering at a similar position are highlighted by red arrows. The superposition of *Mt*FRH dimer with one dimeric unit of the dodecamer shows only minor deviations (rmsd of 0.65 Å for 1,475-Cα aligned).

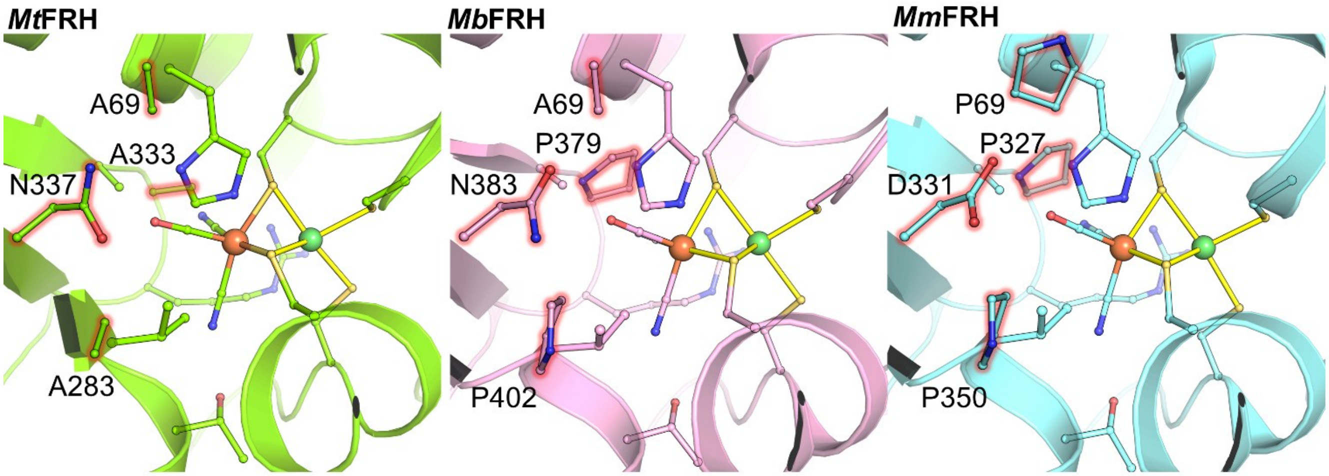

Figure S8. Details of the first and second coordination shells of the active site in FRH structures. The first coordination sphere of the [NiFe]-centre is perfectly conserved among structurally characterised FRHs, but differences can be seen in the second coordination sphere. The positions 283 and 333 (*Mt*FRH numbering) in the vicinity of the iron atom are occupied by prolines in *Mm*FRH and *Mb*FRH, being in *Mt*FRH replaced by alanines, a less rigid residue. Positions 69 and 337 are occupied by an alanine and an asparagine, respectively, as in *Mb*FRH, but are different from those in *Mm*FRH.

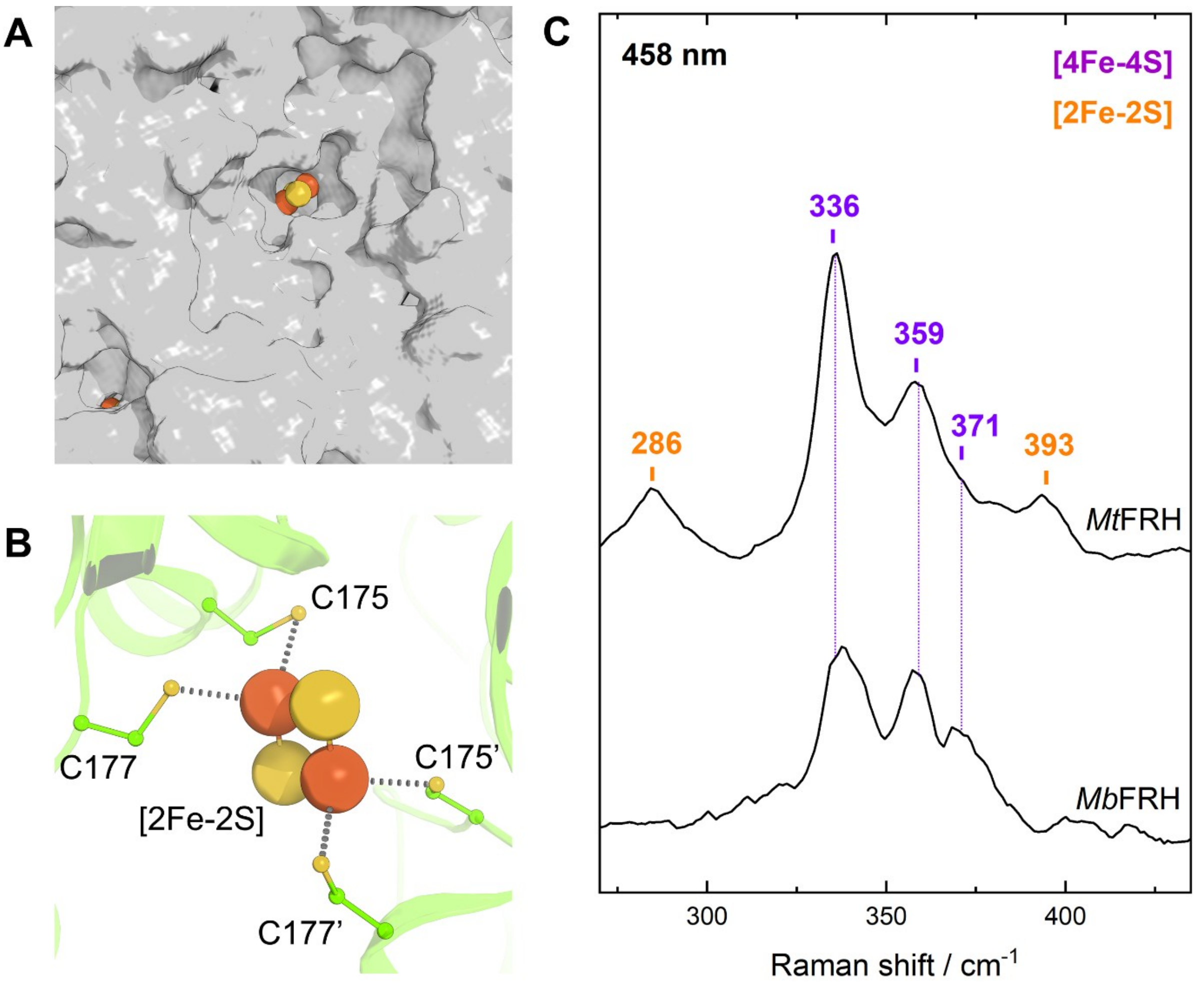

Figure S9. Coordination environment and redox state of the [2Fe-2S] cluster at the dimeric interface of *Mt*FRH^{dimer3}. A) The [2Fe-2S] cluster is directly exposed to the solvent, as shown by the protein surface coloured in grey. B) Two cysteines from each FRHG protomer coordinate the [2Fe-2S] cluster. The two cysteines of the dimeric partner are labelled with a prime (′). C) RR spectra from FRH crystals to determine the redox states of the [4Fe-4S] and [2Fe-2S] clusters in *Mt*FRH in comparison to *Mb*FRH, normalised to the intensity of the band of phenylalanine at 1004 cm^{-1}.[4, 5] Spectral contributions from the FeS clusters are more pronounced and also exhibit some other features compared to *Mb*FRH, suggesting a larger amount of oxidised species in *Mt*FRH. Additional bands at 286 and 393 cm^{-1} can be assigned to the oxidised protomer-bridging [2Fe-2S] cluster. The [2Fe-2S] centre has been proposed to enable the distribution of electrons among individual protomers of the dodecameric enzyme, which was corroborated by EPR experiments on *Mb*FRH.[6] Indeed, the [2Fe-2S] cluster connects the two electron transfer chains in a dimer, which would otherwise be separated by at least 17 Å, thereby likely allowing cooperativity in the complexes.

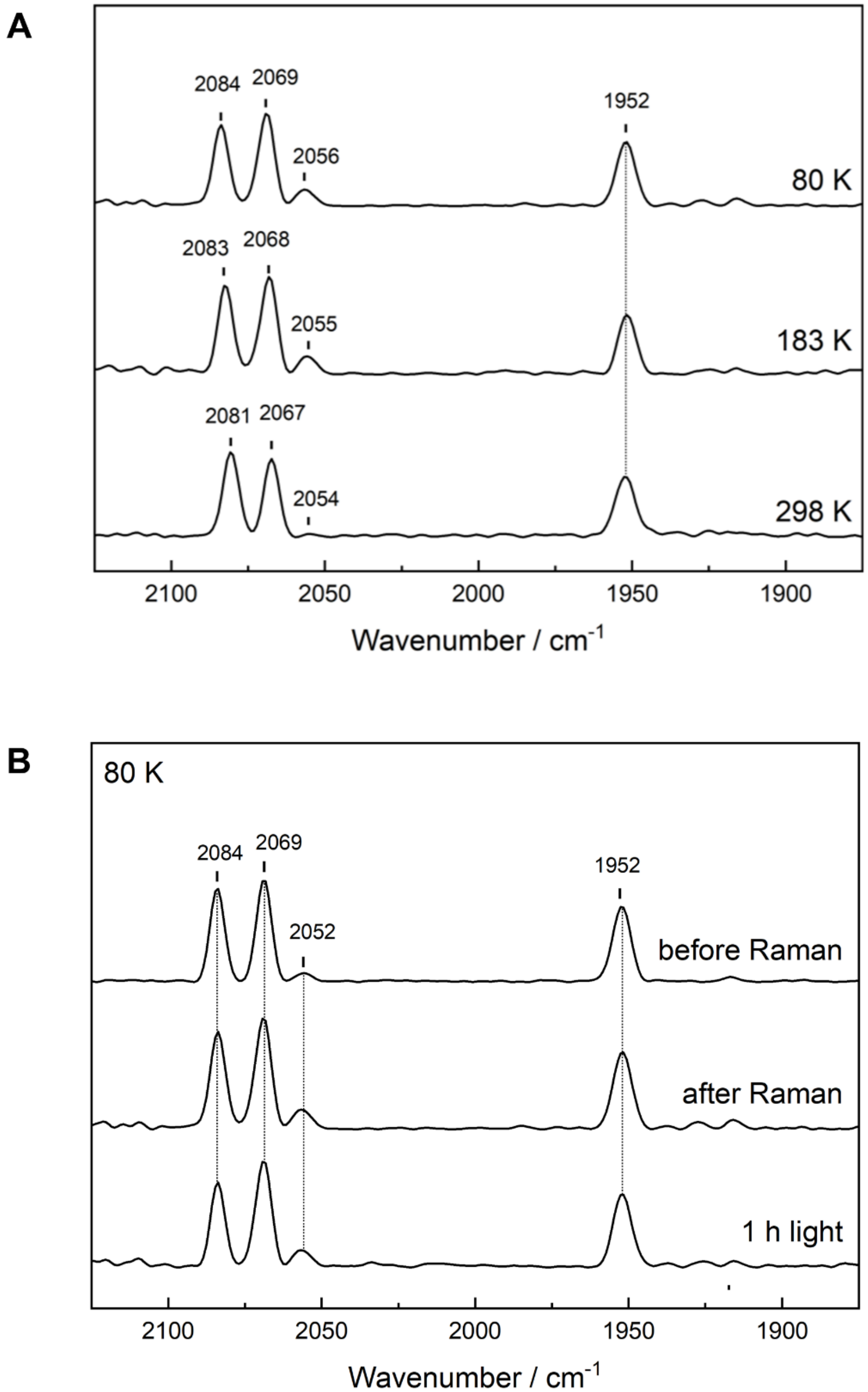

Figure S10. Temperature- and illumination-dependent IR on *Mt*FRH crystals. A) Temperature-dependent IR spectra from 80 K to room temperature. The position of the CO mode changes marginally, while the CN vibrations shift ca. 2-3 cm^{-1} to a lower frequency when increasing the temperature. B) IR spectra before (top) and after (middle) the Raman measurement at 568 nm and after one hour illumination with a focused power LED at 590 nm. No significant spectral changes were observed.

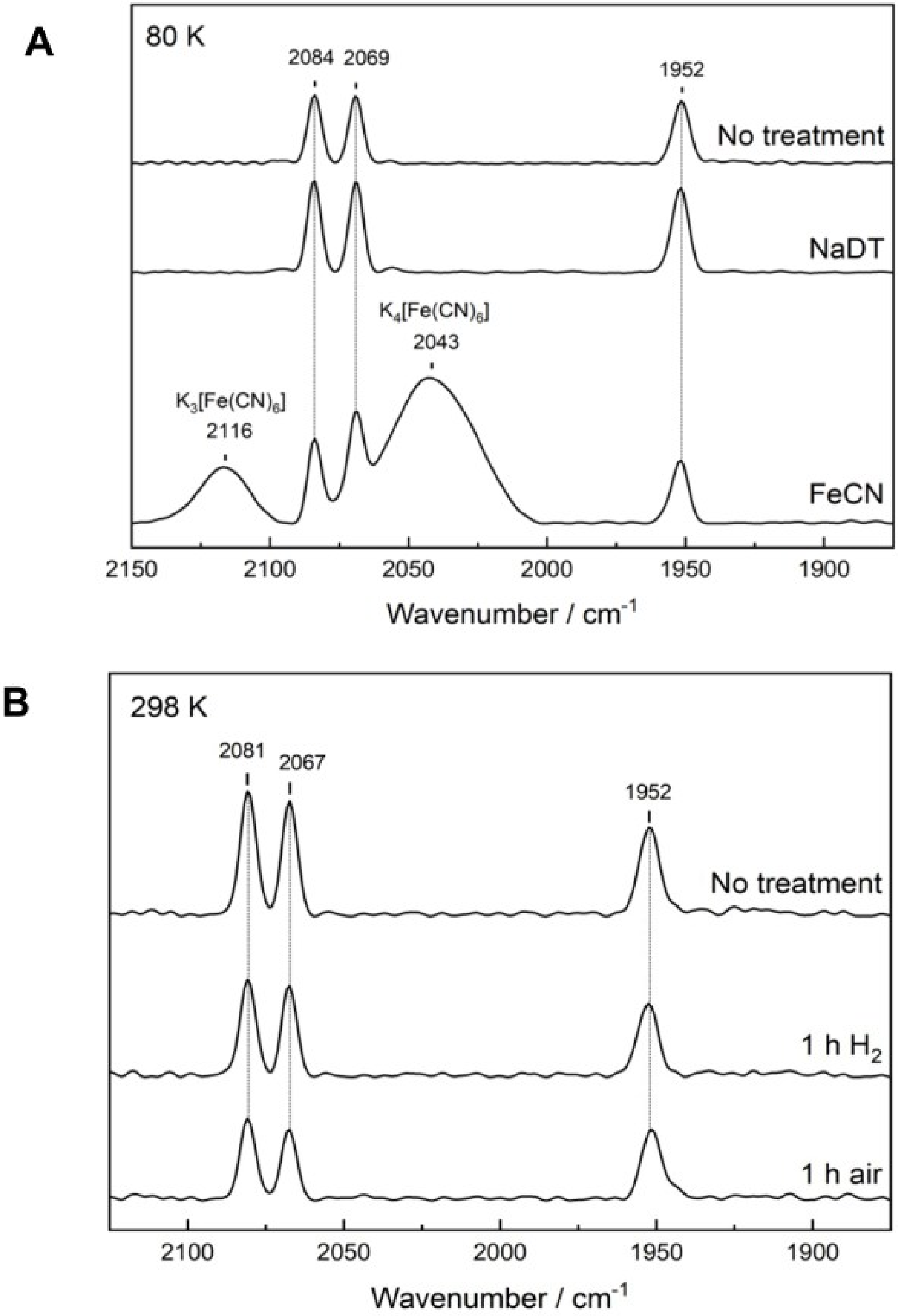

Figure S11. IR spectra of *Mt*FRH crystals under different redox conditions. A) Spectra recorded at 80 K with and without soaking in sodium dithionite (NaDT) or ferricyanide (FeCN). B) Spectra recorded at 298 K without treatment and after purging with H_2 or synthetic air. No significant spectral changes were observed.

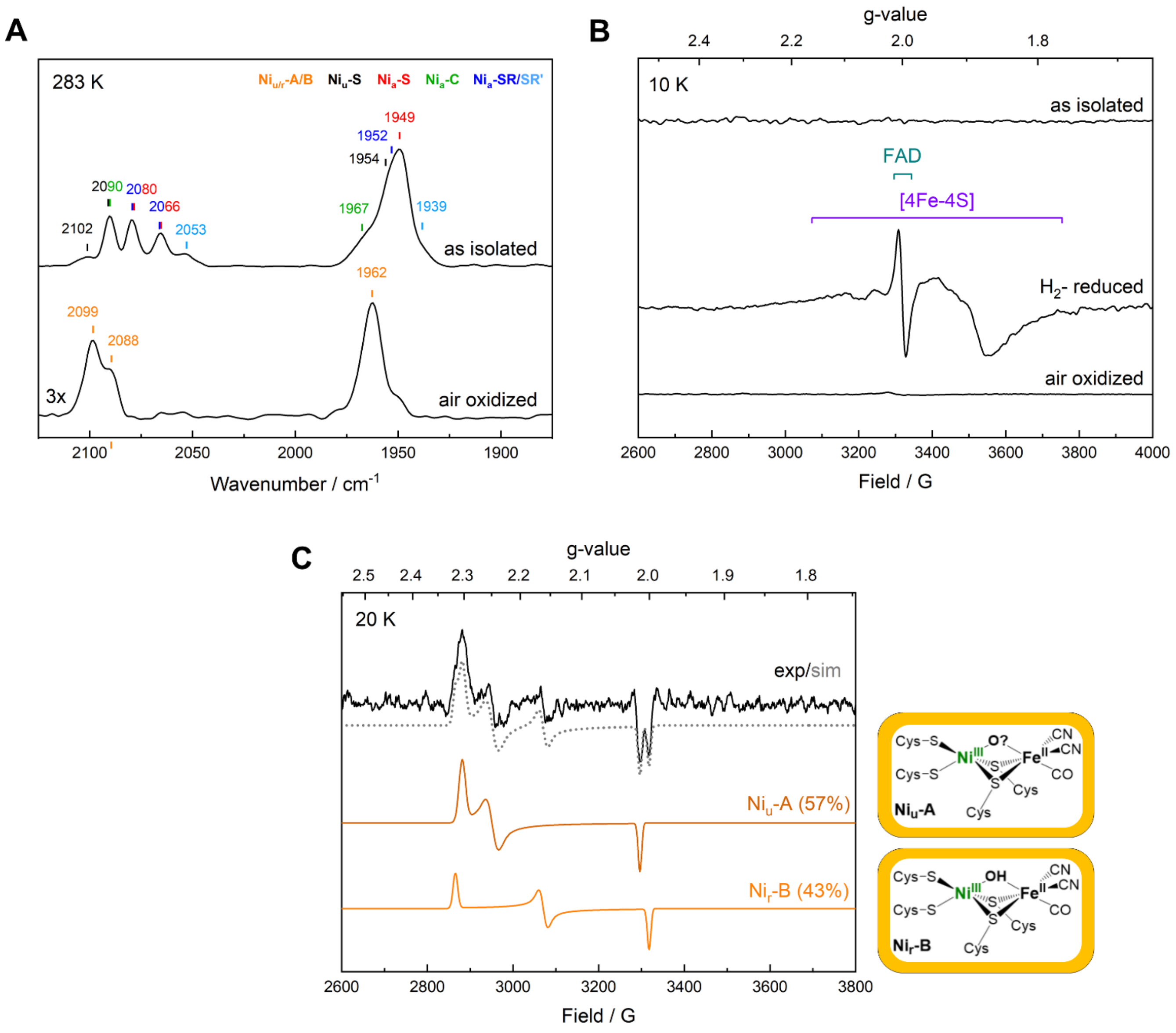

Figure S12. EPR and IR spectra of *Mt*FRH solution. A) IR spectra recorded at 283 K of the as-isolated state and after H_2-reduction, followed by fast air reoxidation. B) EPR spectra after different redox treatments recorded at 10 K. The as-isolated sample remains EPR silent, indicating a fully oxidised iron-sulfur cluster chain and FAD. After exposure to H_2, distinct signals related to [4Fe-4S] clusters and a semiquinone radical can be observed. This verifies that the enzymes can be reduced by H_2, without the need for the other substrate F_{420}. After reoxidation with air, the iron-sulfur clusters and FAD are again EPR silent due to their diamagnetic oxidised nature. C) EPR spectrum after reoxidation with air recorded at 20 K. The typical rhombic signature of the Ni_u-A and the Ni_r-B state of the active site could be deconvoluted in the reoxidised sample by simulations.

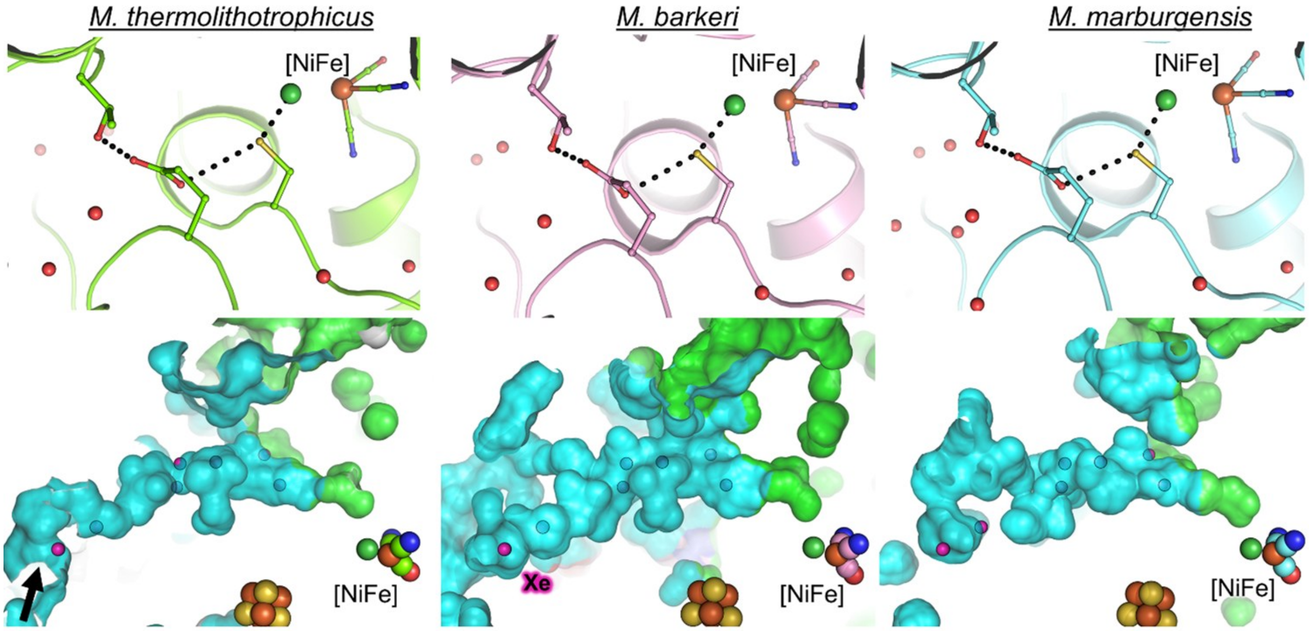

Figure S13. Proposed proton transfer (upper row) and gas channels (lower row) in the structurally characterised group 3 [NiFe]-hydrogenases. Upper row: The proposed proton transfers according to Tai et al.[14] *Mt*FRH is shown in green cartoon, *Mb*FRH in pink (PDB 6QGR) and *Mm*FRH in cyan (PDB 4OMF). The cysteine and glutamate that are proposed to contribute to the proton transfer to the [NiFe]-centre are shown in sticks, water molecules are represented as red spheres, and the metals of the [NiFe]-centre are depicted as spheres with their ligands shown as sticks. Lower row: The proposed gas channels based on the channel identified via xenon derivatisation experiments in *Mb*FRH[6]. The pink spheres correspond to xenon atoms, which were superposed to the *Mt*FRH^{dimer1} and *Mm*FRH structures to predict their hydrophobic gas channels. Cyan and blue surfaces correspond to FRHA and FRHG, respectively. Cofactors are shown in spheres, and the carbon, nitrogen, oxygen, sulfur, iron, and nickel atoms are coloured green/pink/cyan, blue, red, yellow, orange and dark green, respectively.

References

(1) Long, M.; Liu, J.; Chen, Z.; Bleijlevens, B.; Roseboom, W.; Albracht, S. P. J. Characterization of a HoxEFUYH type of [NiFe]-hydrogenase from *Allochromatium vinosum* and some EPR and IR properties of the hydrogenase module. *J. Biol. Inorg. Chem.* **2007**, *12* (1), 62-78. DOI: 10.1007/s00775-006-0162-1.
(2) Germer, F.; Zebger, I.; Saggu, M.; Lendzian, F.; Schulz, R.; Appel, J. Overexpression, isolation, and spectroscopic characterization of the bidirectional [NiFe]-hydrogenase from *Synechocystis* sp. PCC 6803. *J. Biol. Chem.* **2009**, *284* (52), 36462-36472. DOI: 10.1074/jbc.M109.028795.
(3) Horch, M.; Lauterbach, L.; Mroginski, M. A.; Hildebrandt, P.; Lenz, O.; Zebger, I. Reversible active site sulfoxygenation can explain the oxygen tolerance of a NAD^+-reducing [NiFe]-hydrogenase and its unusual infrared spectroscopic properties. *J. Am. Chem. Soc.* **2015**, *137* (7), 2555-2564. DOI: 10.1021/ja511154y.
(4) Preissler, J.; Wahlefeld, S.; Lorent, C.; Teutloff, C.; Horch, M.; Lauterbach, L.; Cramer, S. P.; Zebger, I.; Lenz, O. Enzymatic and spectroscopic properties of a thermostable [NiFe]-hydrogenase performing H_2-driven NAD^+-reduction in the presence of O_2. *Biochim. Biophys. Acta Bioenerg.* **2018**, *1859* (1), 8-18. DOI: 10.1016/j.bbabio.2017.09.006.
(5) Kulka-Peschke, C. J.; Schulz, A.-C.; Lorent, C.; Rippers, Y.; Wahlefeld, S.; Preissler, J.; Schulz, C.; Wiemann, C.; Bernitzky, C. C. M.; Karafoulidi-Retsou, C.; et al. Reversible glutamate coordination to high-Valent nickel protects the active site of a [NiFe]-hydrogenase from oxygen. *J. Am. Chem. Soc.* **2022**, *144* (37), 17022-17032. DOI: 10.1021/jacs.2c06400.
(6) Ilina, Y.; Lorent, C.; Katz, S.; Jeoung, J.-H.; Shima, S.; Horch, M.; Zebger, I.; Dobbek, H. X-ray crystallography and vibrational spectroscopy reveal the key determinants of biocatalytic dihydrogen cycling by [NiFe]-hydrogenases. *Angew. Chem. Int. Ed Engl.* **2019**, *58* (51), 18710-18714. DOI: 10.1002/anie.201908258.
(7) Greene, B. L.; Vansuch, G. E.; Wu, C.-H.; Adams, M. W. W.; Dyer, R. B. Glutamate Gated Proton-Coupled Electron Transfer Activity of a [NiFe]-Hydrogenase. *Journal of the American Chemical Society* **2016**, *138* (39), 13013-13021. DOI: 10.1021/jacs.6b07789.
(8) Lubitz, W.; Ogata, H.; Rüdiger, O.; Reijerse, E. Hydrogenases. *Chemical Reviews* **2014**, *114* (8), 4081-4148. DOI: 10.1021/cr4005814.
(9) Horch, M.; Schoknecht, J.; Mroginski, M. A.; Lenz, O.; Hildebrandt, P.; Zebger, I. Resonance Raman spectroscopy on [NiFe]-hydrogenase provides structural insights into catalytic intermediates and reactions. *J. Am. Chem. Soc.* **2014**, *136* (28), 9870-9873. DOI: 10.1021/ja505119q.
(10) Siebert, E.; Rippers, Y.; Frielingsdorf, S.; Fritsch, J.; Schmidt, A.; Kalms, J.; Katz, S.; Lenz, O.; Scheerer, P.; Paasche, L.; et al. Resonance Raman Spectroscopic Analysis of the [NiFe] Active Site and the Proximal [4Fe-3S] Cluster of an O_2-Tolerant Membrane-Bound Hydrogenase in the Crystalline State. *The Journal of Physical Chemistry B* **2015**, *119* (43), 13785-13796. DOI: 10.1021/acs.jpcb.5b04119.
(11) Albracht, S. P. J. Nickel hydrogenases: in search of the active site. *Biochimica et Biophysica Acta (BBA) - Bioenergetics* **1994**, *1188* (3), 167-204. DOI: 10.1016/0005-2728(94)90036-1.
(12) Teixeira, M.; Moura, I.; Xavier, A. V.; Huynh, B. H.; DerVartanian, D. V.; Peck, H. D.; LeGall, J.; Moura, J. J. Electron paramagnetic resonance studies on the mechanism of activation and the catalytic cycle of the nickel-containing hydrogenase from *Desulfovibrio gigas*. *Journal of Biological Chemistry* **1985**, *260* (15), 8942-8950. DOI: 10.1016/S0021-9258(17)39440-1.
(13) Edgar, R. C. MUSCLE: a multiple sequence alignment method with reduced time and space complexity. *BMC Bioinformatics* **2004**, *5*, 113. DOI: 10.1186/1471-2105-5-113.
(14) Tai, H.; Nishikawa, K.; Higuchi, Y.; Mao, Z. W.; Hirota, S. Cysteine SH and Glutamate COOH Contributions to [NiFe]-hydrogenase Proton Transfer Revealed by Highly Sensitive FTIR Spectroscopy. *Angewandte Chemie (International ed. in English)* **2019**, *58* (38), 13285-13290. DOI: 10.1002/anie.201904472 From NLM.
